# Chromosome 7p linkage and association study for diabetes related traits and type 2 diabetes in an African-American population enriched for nephropathy

**DOI:** 10.1186/1471-2350-11-22

**Published:** 2010-02-08

**Authors:** Tennille S Leak, Carl D Langefeld, Keith L Keene, Carla J Gallagher, Lingyi Lu, Josyf C Mychaleckyj, Stephen S Rich, Barry I Freedman, Donald W Bowden, Michèle M Sale

**Affiliations:** 1 Center for Human Genomics, Wake Forest University School of Medicine, Winston-Salem, NC, USA; 2 Department of Epidemiology, Graduate School of Public Health, University of Pittsburgh, Pittsburgh, Pennsylvania, USA; 3 Department of Biostatistical Sciences, Wake Forest University School of Medicine, Winston-Salem, NC, USA; 4 Center for Public Health Genomics, University of Virginia, Charlottesville, VA, USA; 5 Department of Public Health Sciences, University of Virginia, Charlottesville, VA, USA; 6 Department of Biochemistry, Wake Forest University School of Medicine, Winston-Salem, NC, USA; 7 Milton S Hershey Medical Center, Pennsylvania State University, Hershey, PA, USA; 8 Department of Biochemistry and Molecular Genetics, University of Virginia, Charlottesville, VA, USA; 9 Department of Internal Medicine, Wake Forest University School of Medicine, Winston-Salem, NC, USA; 10 Department of Medicine, University of Virginia, Charlottesville, VA, USA

## Abstract

**Background:**

Previously we performed a linkage scan of 638 African American affected sibling pairs (ASP) with type 2 diabetes (T2D) enriched for end-stage renal disease (ESRD). Ordered subset linkage analysis (OSA) revealed a linkage peak on chromosome 7p in the subset of families with earlier age of T2D diagnosis.

**Methods:**

We fine mapped this region by genotyping 11 additional polymorphic markers in the same ASP and investigated a total of 68 single nucleotide polymorphisms (SNPs) in functional candidate genes (*GCK1, IL6, IGFBP1 *and *IGFBP3) *for association with age of T2D diagnosis, age of ESRD diagnosis, duration of T2D to onset of ESRD, body mass index (BMI) in African American cases and T2D-ESRD in an African American case-control cohort. OSA of fine mapping markers supported linkage at 28 cM on 7p (near D7S3051) in early-onset T2D families (max. LOD = 3.61, P = 0.002). SNPs in candidate genes and 70 ancestry-informative markers (AIMs) were evaluated in 577 African American T2D-ESRD cases and 596 African American controls.

**Results:**

The most significant association was observed between ESRD age of diagnosis and SNP rs730497, located in intron 1 of the *GCK1 *gene (recessive T2D age-adjusted *P *= 0.0006). Nominal associations were observed with *GCK1 *SNPs and T2D age of diagnosis (BMI-adjusted *P *= 0.014 to 0.032). Also, one *IGFBP1 *and four *IGFBP3 *SNPs showed nominal genotypic association with T2D-ESRD (*P *= 0.002-0.049). After correcting for multiple tests, only rs730497 remanined significant.

**Conclusion:**

Variant rs730947 in the *GCK1 *gene appears to play a role in early ESRD onset in African Americans.

## Background

A genome wide linkage scan was performed on 638 African American affected sibling pairs (ASPs) with type 2 diabetes (T2D) from 247 families; 166 families contained at least one ASP concordant for diabetic end-stage renal disease (T2D-ESRD) [1]. Ordered subset analysis (OSA) revealed a linkage peak on chromosome 7p in the subset of T2D families with an early age of diagnosis (29% of pedigrees, max. LOD = 3.85, P = 0.003 for the change in LOD score) [1]. T2D-ESRD subsets with lower body mass index (BMI) (64% of pedigrees, max. LOD = 3.93, P = 0.010) and longer duration from T2D diagnosis to ESRD onset (37% of pedigrees, max. LOD = 3.59, P = 0.010) also showed evidence for linkage at this locus [2].

Fine mapping of this region using the same African American families was undertaken in order to localize the peak and to determine the primary phenotype of association. There are several plausible diabetes and nephropathy candidate genes in the resulting region of interest, including glucokinase isoform 1 (*GCK1)*, interleukin-6 (*IL6)*, insulin growth factor binding protein 1 (*IGFBP1) *and insulin growth factor binding protein 3 (*IGFBP3)*.

Mutations of *GCK *have been identified in subjects with maturity-onset diabetes of the Young (MODY) [3,4]. However, single nucleotide polymorphisms (SNPs) in this gene have also been shown to be associated with typical T2D [5-7]. A dinucleotide (CA)_n _repeat element located approximately 10 kb 3' to the coding region has also shown association with T2D across several diverse ethnic populations [8-10].

The cytokine interleukin 6 (IL-6) is an essential regulator of the acute phase response associated with T2D and diabetic nephropathy [11,12]. Variants in the *IL6 *promoter region have been shown to affect promoter strength [13].

The insulin like growth factors 1 and 2 circulate tightly bound to IGFBP-1 and IGFBP-3. A cross-sectional study of T2D cases and controls found that polymorphisms in *IGFBP3 *was associated with levels of HbA_1c _[14]. Also, *in vivo *and *in vitro *studies have demonstrated that IGFBP-3 is a potent insulin antagonist [15].

We fine mapped the region of interest using additional microsatellite markers, and investigated associations with SNPs in four plausible candidate genes with age of T2D diagnosis, age of ESRD diagnosis, duration of T2D to onset of ESRD, BMI and T2D-ESRD in an African American cohort.

## Methods

### Selection criteria and recruitment of African American families

This study was conducted under Institutional Review Board approval from Wake Forest University School of Medicine and adhered to the tenets of the Declaration of Helsinki. All participants provided written informed consent. Clinical characteristics and recruitment of African American patients have been described previously [1,2]. DNA samples were collected from self-described African American families with multiple T2D affected members. Briefly, families were originally identified through a proband with impaired renal function associated with T2D. Medical records were reviewed to verify the etiology of the nephropathy. Impaired renal function was attributed to diabetes in the presence of the following criteria: serum creatinine ≥ 1.5 mg/dl, diabetes for >10 years or presence of proliferative diabetic retinopathy, and/or proteinuria ≥ 500 mg/24 h or >100 mg/dl, in the absence of other known causes of renal failure. T2D was diagnosed in patients developing diabetes after the age of 35 years and treated at the time of recruitment with oral hypoglycemic agents, insulin, or diet and exercise, where treatment was considered permanent (i.e., excluding steroid-induced diabetes and gestational diabetes). The family set for the genome-wide scan comprised 247 African American families with 638 ASPs, totaling 675 individuals.

### Case-control subjects

Identification, clinical characteristics, and recruitment of African Americans and European American patients and controls have been described previously [16]. Briefly, 577 unrelated African American patients with T2D, born in North Carolina, South Carolina, Georgia, Tennessee or Virginia, were recruited from dialysis facilities. T2D was diagnosed if patients reported an initial diagnosis of diabetes after 35 years of age, received dietary therapy or hypoglycemic agents in the absence of insulin alone for at least 1 year after diagnosis, and were currently receiving diabetes medications. Case subjects had severe diabetes accompanied by nephropathy, and T2D at least 5 years prior to initiating renal replacement therapy, background or greater diabetic retinopathy and/or > 3+ proteinuria on urinalysis in the absence of other causes of nephropathy. Nondiabetic control subjects were recruited from the same geographic region: 596 were African American and 39 European American without a prior diagnosis of T2D or renal disease, born in the same southeastern states, were recruited. DNA extraction was performed using the PureGene system (Gentra Systems, Minneapolis, MN). DNA was also obtained from 44 Yoruba Nigerians (YRI) from the National Institute of General Medical Sciences Human Variation Collection (Coriell Cell Repositories, Camden, NJ).

For admixture analyses, 39 unrelated European American controls were recruited using the same criteria as African American controls. DNA was also obtained from 44 YRI from the National Institute of General Medical Sciences Human Variation Collection (Coriell Cell Repositories, Camden, NJ).

### Genotyping for fine mapping

Eleven fine mapping microsatellites at an average spacing of 4 cM were selected from the Marshfield Center of Medical Genetics database http://research.marshfieldclinic.org/genetics/ and the Genome Database http://www.gdb.org/gdb/. The polymerase chain reaction (PCR) was used to incorporate fluorescently labeled primers from Integrated DNA Technologies, Inc. (Coralville, IA) and Applied Biosystems (Foster City, CA) into PCR products, which were then electrophoresed on an ABI PRISM 3700 DNA Analyzer and results analyzed with Genescan Analysis Software (Applied Biosystems, Foster City, CA). Electropherograms were imported into GeneMapper (Applied Biosystems, Foster City, CA) for binning and allele calling. Familial relationships and Mendelian inconsistencies were checked using Prest [17] and PedCheck [18]. Inconsistent familial relationships were discarded from further analysis.

### Ordered subsets linkage analysis

A series of OSA [19] were computed to investigate the influence of a pedigree's mean age of diagnosis, BMI and duration of T2D to ESRD onset. The statistical significance of the change in the LOD score was evaluated by a permutation test under the null hypothesis that the ranking of the covariate is independent of the LOD score of the family on the target chromosome. Thus, the families were randomly permuted with respect to the covariate ranking, and an analysis proceeded as above for each permutation of these data. The resulting empirical distribution of the change in the LOD scores yielded a chromosome-wide *P *value (Δ*P*).

### Diabetes candidate gene selection and genotyping

**Figure 1:**
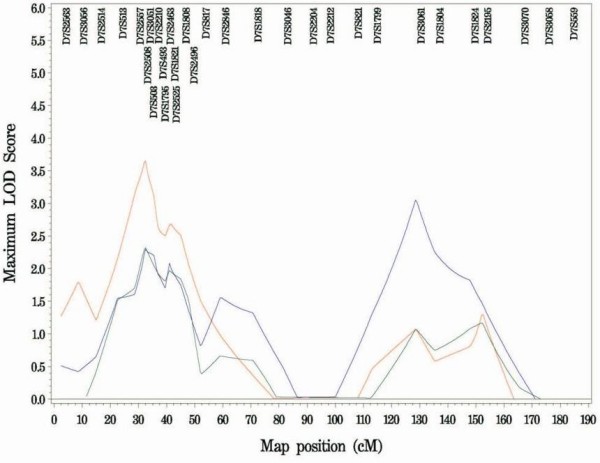
**Chromosome 7p Ordered Subset Analysis**. Chromosome 7p fine mapping linkage results showing the Ordered Subset Analyses of early age at type 2 diabetes diagnosis (red), lower body mass index (green) and longer duration of type 2 diabetes mellitus to development of end stage renal disease (blue).

Plausible functional candidate genes (*IL6*, *GCK1*, *IGFBP1 *and *IGFBP3) *that lie under the intersection of the optimal linkage peaks for early age of T2D, lower BMI and longer duration to ESRD was selected (Figure 1). This regions roughly spans the distance between D7S513 and D7S1818. In the absence of a comprehensive African American SNP database of allele frequencies, we used the genotypic data of the YRI and CEPH Europeans (CEU) from the International HapMap project (HapMap #20, NCBI B35 assembly). Using the largest gene transcript plus 5 kb upstream and downstream of the gene, we selected YRI HapMap SNPs with a minor allele frequency (MAF) ≤ 0.05, excluded SNPs with an Illumina designability score < 1.0, then used the aggressive tagging option of Tagger [20] implemented in Haploview 3.2 [21]. Next, we selected CEU HapMap SNPs with MAF ≤ 0.05, forced the exclusion of Illumina-undesignable SNPs and inclusion of the already selected YRI tagSNPs, then used the aggressive tagging option of Tagger [20] to select any additional tags required to capture CEU variation. In addition, we added previously associated SNPs from the literature, yielding a total of 68 SNPs: glucokinase isoform 1 (*GCK1*, 24 SNPs); interleukin-6 (*IL6*, 17 SNPs); insulin growth factor binding protein 1 (*IGFBP1*, 16 SNPs) and insulin growth factor binding protein 3 (*IGFBP3*, 11 SNPs).

Genotyping was performed by Illumina Genotyping Services (Illumina Inc., San Diego, CA). Six SNPs were genotyped using iPlex methodology on a MassARRAY system (Sequenom Inc., San Diego, CA) [22]: *IL6 *rs1800797; *GCK1 *rs741038, rs2971672 and *IGFBP1: *rs1496495, rs1908751, rs9658239. Primer sequences are available on request. The genotyping success rates were >96.5 in cases and >96.1% in controls. For quality control purposes each plate contained 2 duplicate samples and 4 inter-plate controls. Concordance between duplicate samples included in the genotyping was >98.9%.

### Genotyping for admixture analyses

Seventy biallelic Admixture Informative Markers (AIMs), selected to maximize European and African allele frequency differences and sample all non-acrocentric arms of the autosomal genome, were genotyped by Illumina Inc.'s Custom Genotyping Service (Illumina Inc., San Diego, CA) in 577 African American cases, 596 African American controls, 44 YRI and 39 European Americans. Primer sequences are available on request. Genotyping success rates for AIMS were >97.4% in African American cases and >95.6% in African American controls.

### Statistical Analysis

Characteristics were compared and tested for significant differences using χ^2 ^tests for categorical variables, and one-way ANOVA test for continuous variables. Hardy-Weinberg equilibrium (HWE) was assessed using the χ^2 ^goodness-of-fit statistic at a significance level of ≤ 0.01. Haplotype block structure was established using Haploview 3.2 [21], using the block definition from Gabriel *et al*. [23].

Tests of association under the three *a priori *genetic tests of association (dominant, additive and recessive) are reported. Due to a lack of validity of the large sample χ^2 ^test statistic, only the dominant model was considered for SNPs with ten or fewer individuals that were homozygous for the minor allele.

Quantitative trait (duration of T2D to ESRD onset, BMI, age at T2D and ESRD onset) association analysis for all SNPs was performed using a series of analysis of variance (ANOVA) tests implemented in QSNPGWA [24].

SNPs that showed nominal evidence for association with age of T2D onset were adjusted by BMI. Additionally, ESRD age, duration of T2D to ESRD onset and BMI were adjusted for age of T2D onset. All analyses were adjusted using linear regression on *a priori *genotypic models, and conducted using Stata 10 (College Station, TX).

Unadjusted odds ratios (ORs) and 95% confidence intervals (CI) in the case-control population were computed using SNPGWA to test all SNPs for genotypic association with T2D-ESRD [25]. SNPs that showed nominal evidence for association were then tested using a permutation test (1,000 permutations) as implemented in SNPGWA.

Individual ancestral proportions were calculated using an expectation-maximization (EM) algorithm (FRAPPE) [26] under a two-population model. Logistic regression tests of genetic models included adjustments for individual estimates of African ancestry, implemented in the program SNPADMIX [25].

PS power and sample size program specifying a two-sided test at an alpha level of ≤ 0.05, to detect an OR of 1.30 was utilized to approximate study power [27].

We report nominally significant associations at the *P *≤ 0.05 level. To correct for multiple tests at the gene level the conservative Bonferonni method was used, with a *P*-value ≤ 0.0007 considered significant evidence for association when assuming independence based on LD.

## Results

### Fine mapping linkage analyses

OSA including the new fine mapping microsatellite data continued to support linkage at 28 cM on chromosome 7p, closest to D7S3051, in early-onset T2D families. Twenty-one percent of the pedigrees account for a maximum LOD = 3.61 (*P *= 0.002) with a mean age at diagnosis 29.5 ± 3.0 years vs. mean age at diagnosis of the unlinked families 44.5 ± 7.3. Although the max. LOD score dropped (from 3.85, *P *= 0.003 in the genome scan) the LOD-1 interval increased from 11.5-38.2 cM to 11.5-49.5 cM. There was no significant difference (*P *= 0.56) between mean BMI in the linked early-onset families (31.5 kg/m^2 ^± 7.62) versus the unlinked (31.8 kg/m^2 ^± 7.51) pedigrees. The evidence for linkage in subsets of T2D-ESRD families with lower BMI (64% of pedigrees; max. LOD = 2.20, *P *= 0.11) and longer duration of diabetes before onset of ESRD (56% of pedigrees; max. LOD = 2.81, *P *= 0.20) was no longer significant (Figure 1).

### Case-control population

Characteristics of the study population used for tests of association with positional candidate genes are shown in Table 1. The mean age of examination for the non-diabetic controls is 7.5 years later than the mean age of onset for diabetes in the T2D-ESRD participants. The estimated mean proportion of African ancestry in the case subjects was 81.7 ± 13.3 in T2D-ESRD cases and 79.1 ± 13.1 in controls (*P *= 0.0008).

**Table 1:** Characteristics of African American subjects

Trait	T2D-ESRD Cases		Controls	
	n*		n*	
% Female (n)	577	61% (352)	596	51% (306)
Age at exam (years ± SD)	541	62.2 ± 10.3‡	448	49.3 ± 9.8
Age at T2D diagnosis (years ± SD)	544	41.8 ± 11.6	-	-
Age at ESRD diagnosis (years ± SD)	560	59.0 ± 10.5	-	-
BMI (kg/m^2^)	559	29.6 ± 7.0†	450	29.8 ± 7.1
T2D duration to ESRD onset (years ± SD)	511	20.3 ± 10.5	-	-
Mean African Ancestry	577	81.7 ± 13.3‡	596	79.1 ± 13.1

*n = number with data available. ‡ Mean differs significantly between cases and controls P-value < 0.001. †Reflects dry weight on dialysis. All cases were diagnosed with T2D and ESRD.

### Hardy-Weinberg Equilibrium and linkage disequilibrium

All SNPs in candidate genes were examined for departures from HWE assumptions in cases and controls separately. Deviations from HWE (*P *≤ 0.01) were observed for *IL6 *rs1474347 in controls (*P *= 0.002) and *IGFBP1 *rs1496495 in cases (*P *= 0.001). Although inconsistent with HWE proportions, these SNPs were retained for exploratory analyses, however there was no evidence for association of these SNPs with any of the traits investigated.

The 24 *GCK1 *SNPs fell into five blocks of LD, with pairwise values of D' values ranging from 0.41-1.00, and r^2 ^= 0.003-0.76. D' values between the 17 *IL6 *SNPs ranged from 0.34-1.00, with r^2 ^values of 0.0002-0.82, resulting in one high LD block. The 16 *IGFBP1 *SNPs fell within three blocks of high LD, with SNPs in the 3'UTR contained in block 2 (D' = 0.88-0.99; r^2 ^= 0.02-0.34) and the 11 genotyped *IGFBP3 *SNPs fell within two blocks of LD (data not shown).

### Analyses with age at diagnosis of T2D and age at ESRD

Association analyses revealed two *GCK1 *SNPs nominally associated with T2D age at diagnosis and five *GCK1 *SNPs associated with age at ESRD onset (Table 2). The most significant result from the entire dataset was the association with intron 1 SNP rs730497 and age at ESRD onset (*P *= 0.0004; T2D age-adjusted *P *= 0.0006). The homozygote recessive genotype ("*AA*") for *IL6 *SNP rs2069849, located in exon 5, was nominally associated with age at ESRD onset (T2D age-adjusted *P *= 0.013). SNPs in *IGFBP1 *and *IGFBP3 *were not associated with T2D age at diagnosis or ESRD onset (Additional file 1, Table S1 and S2).

**Table 2:** Nominally significant (P < 0.05) single-SNP genotypic tests of association with T2D age at diagnosis, ESRD age at onset, BMI on dialysis, and duration of T2D to ESRD onset.

**Phenotype**	**Gene**	**Marker**	**Major/Minor alleles**	**MAF Case**	**1/1** **Mean ± SD (n)**	**1/2** **Mean ± SD (n)**	**2/2** **Mean ± SD (n)**	**Dominant P-value**	**Additive P-value**	**Recessive P-value**
				
ESRD age	*IL6*	Rs2069849	G/A	0.15	59.0 ± 10.5 (419)	59.8 ± 10.4 (140)	53.7 ± 10.4 (16)	0.896	0.581	**0.040 (0.013)^a^**
Duration of T2D to ESRD	*IL6*	rs1554606	C/A	0.34	20.2 ± 13.7 (255)	19.7 ± 14.7 (245)	15.8 ± 13.0 (68)	0.247	0.052	**0.007 (0.006)^a^**
T2D age	*GCK1*	rs2908296	C/A	0.35	41.6 ± 11.0 (235)	42.8 ± 12.2 (277)	38.0 ± 10.3 (63)	0.733	0.313	**0.007 (0.014)^b^**
T2D age	*GCK1*	rs12673242	A/G	0.37	41.3 ± 11.4 (236)	43.2 ± 11.9 (258)	39.0 ± 11.2 (81)	0.38	0.606	**0.023 (0.032)^b^**
ESRD age	*GCK1*	rs2908296	C/A	0.35	59.2 ± 10.0 (235)	59.4 ± 11.2 (277)	56.1 ± 8.7 (63)	0.632	0.157	**0.027 (0.133)^a^**
ESRD age	*GCK1*	rs2971676	G/A	0.24	59.4 ± 10.4 (328)	59.0 ± 10.8 (217)	54.4 ± 8.8 (30)	0.328	0.092	**0.02 (0.035)^a^**
ESRD age	*GCK1*	rs2971675	C/A	0.23	59.5 ± 10.5 (338)	58.7 ± 10.8 (210)	54.5 ± 8.4 (27)	0.2	0.065	**0.033 (0.029)^a^**
ESRD age	*GCK1*	rs730497	G/A	0.2	58.9 ± 10.4 (367)	60.0 ± 10.6 (180)	51.5 ± 8.6 (25)	0.989	0.206	**0.0004 (0.0006)^a^**
ESRD age	*GCK1*	rs2908289	G/A	0.27	58.5 ± 10.5 (315)	60.6 ± 10.7 (211)	55.1 ± 8.8 (49)	0.223	0.827	**0.008 **(0.129)^a^
BMI	*IGFBP1*	rs1908751	C/T	0.33	29.8 ± 7.3 (254)	29.2 ± 6.7 (243)	31.6 ± 7.1 (60)	0.838	0.436	**0.043 **(0.067)^a^
BMI	*IGFBP3*	rs10255707	G/A	0.04	29.9 ± 7.1 (511)	27.3 ± 5.4 (33)	23.5 ± 7.4 (2)	**0.022 **(0.120)^a^	-	-
Duration of T2D to ESRD	*IGFBP3*	rs3110697	G/A	0.35	21.3 ± 15.7 (234)	18.5 ± 12.7 (270)	19.8 ± 14.2 (63)	**0.027 **(0.067)^a^	0.055	0.586

Test models refer to the minor allele. *Only the dominant model was considered where the minor allele homozygote count was <10. **Bold**: P-values < 0.05. Unadjusted *P *values without parentheses and *P *values with parentheses adjusted for ^a^T2D age- and ^b^BMI.

### Analyses with duration of T2D to ESRD onset

Association results for duration of T2D to ESRD onset are presented in Additional file 1, Table S3. *IL6 *SNP rs1554606 was nominally associated (T2D age-adjusted *P *= 0.006) with shorter duration of T2D to ESRD onset (Table 2; 15.8 ± 13.0 years for "*AA*" genotype, compared with 20.0 ± 14.1 years for "*CC*"/"*CA*"). Results are further summarized in Table 2.

### Analyses with BMI

Association results for BMI are shown in Table 2. *IGFBP1 *and *IGFBP3 *SNPs were not associated when adjusted for age of T2D diagnosis (*P *value = 0.067 and 0.120 respectively). Single SNP association results for BMI are presented thoroughly in Additional file 1, Table S4.

### Analyses with T2D-ESRD in the African American case-control population

Single SNP genotypic association results (*P *≤ 0.05) for T2D-ESRD are shown in Additional file 1, Table S5. Nominal evidence of association was observed with *IGFBP3*: two promoter SNPs: rs903889 (dominant *P *= 0.014; admixture-adjusted *P*_*a *_= 0.029), rs924140 (additive *P *= 0.019; *P*_*a *_= 0.033) and two SNPs located in intron 3; rs10255707 (dominant *P *= 0.005; *Pa *= 0.01) and rs3110697 (recessive *P *= 0.002; *Pa *= 0.003). Nominal evidence of association was also detected with *IGFBP1 *rs9658233 located in the 3' UTR region. *GCK1 *and *IL6 *SNPs showed no evidence of association with T2D-ESRD (Additional file 1, Table S6). Genotype frequencies and counts for each SNP are shown in Additional file 1, Table S7.

Tagger [20] results indicated that 2- or 3-SNP haplotypes were not required to capture ungenotyped YRI and CEU HapMap SNPs in these genes.

## Discussion

Evidence of linkage on chromosome 7p in families with age of T2D diagnosis, ESRD onset, lower BMI and longer duration to ESRD was detected in an earlier study of African American families [1,2]. These results generated our interest in genotyping additional polymorphic markers to refine the linkage peak and explore positional candidate genes. Fine mapping analyses continued to support evidence for linkage at 7p in a subset of T2D families with an early age of diagnosis. Although this linkage peak does not appear to have been reported in other populations, two recent reports detected modest evidence of linkage to T2D in the same region in African American families from the GENNID Cohort [28] and an African American population from South Carolina [29].

The present study demonstrates that *GCK1 *rs730497 recessive "AA" genotype is strongly associated with early age ESRD onset (Table 2). *GCK1*, a strong functional candidate for T2D, appears to play a role in susceptibility to early age of ESRD in African Americans. We observed an approximately 6 year shorter duration of T2D to ESRD and early age of T2D onset with recessive *"AA" *homozygotes compared with *"GG" *homozygotes and heterozygotes (Additional file 1, Tables S1 and S3). Earlier reports suggest that nephropathy is rarely obeserved in persons with *GCK1 *mutations [30]. MODY is characterized as non-ketoticT2D that is inherited by an autosomal dominant manner [31-33], with early onset (usually before the age of 25). MODY is often asymptomatic mild hyperglycemia, which can go undetected for prolonged periods of time which can lead to diabetic complication later in adulthood. Hence, it is plausible that in the presence of hyperglycemia and increased BMI (mean BMI 30.8 kg/m^2 ^± 7.1; Additional file 1, Table S4) along with the rs730497 recessive genotype results in ESRD progression. In addition, several reports have shown that MODY genes segregate in late onset T2D cases and families [34-36]. In an effort to reduce the likelihood of including potential MODY2 patients, only individuals with T2D onset over the age of 35 were ascertained as probands.

Recently, Paré *et al*. [37] reported that variant rs730497 is associated with an increase of 0.03% HbA_1c _levels (*P *= 2.8 × 10^-12^) in non-diabetic European Americans from the Women's Genome Health Study (WGHS) and participants from the Boston metropolitan area. The MAF of the rs730497 A "risk"allele was similar in African American cases (0.20) and previously reported European Americans from WGHS (0.17) [37]. Several studies have shown a strong correlation between increased levels of HbA_1c _and nephropathy [38]. The relationship between rs730497 minor "*A*" allele and ESRD progression is unknown, however, marker rs730497 is located 4.8 kb from exon 1 and 30.7 kb from exon 2. The slightly more common *G *allele appears to be ancestral since it is present in the chimpanzee sequence.

Nominal associations with MODY gene *GCK1 *was also observed with T2D age at diagnosis (Table 2). Four *GCK1 *SNPs were found to be associated with T2D risk (recessive model: OR ranging from 1.36-1.87) in a case-control population from Finland [39]. Bonnycastle *et al*. [39] observed an 87% increased T2D risk with marker rs882020. Marker rs2908296 is approximately 14.7 kb from rs882020; it is plausible that these variants may tag or be in LD with the causal variant(s). The reported associated SNPs (rs2284769, rs12534623, rs2268573 and rs882020) [39] and the dinucleotide repeat (D7S531) in Mauritian Creoles [8], African Americans [9], and South Indians [10], were not directly investigated in our African American population due to our HapMap-coverage based SNP selection process, however the 24 *GCK1 *SNPs genotyped captured all 32 HapMap GCK1 SNPs with MAF > 5% with r^2 ^values > 0.80.

*IL6 *SNP rs2069849, located in exon 5, was nominally associated with earlier age of ESRD onset. This variant causes a synonymous coding change for phenylalanine at position 201 (F201), which may alter mRNA stability and translation. Promoter polymorphisms rs1800795 and rs1800796 have shown association with T2D and diabetic nephropathy respectively [40-42]. We investigated these two SNPs in our African American population (Additional file 1, Table S6) and, consistent with a study conducted in a larger population of T2D cases and non-T2D controls [43], did not observe association with T2D-ESRD. The MAF for promoter SNPs rs1800797, rs1800796 and rs1800795 in African American (controls: 0.07 0.09; 0.07, respectively; cases 0.05; 0.11; 0.06, respectively) are comparable with the YRI frequencies (MAF = 0.0; 0.09; 0.0), but contrast with those of the European American controls (MAF = 0.48; 0.04; 0.53). The lack of association may be due to the reduced prevalence of the risk alleles in our African American population.

Modest associations with T2D-ESRD were also detected with one *IGFBP1 *(rs9658233) and four *IGFBP3 *SNPs (rs10255707, rs3110697, rs924140 and rs903889). The major "T" allele for SNP rs9658233, located in the downstream region of *IGFBP1*, appears to confer risk with T2D-ESRD, with OR between 1.25-1.35 (Additional file 1, Table S5). In contrast, having at least one copy of the minor allele for *IGFBP3 *variants rs3110697 and rs924140 appear to protect against T2D-ESRD, with OR between 0.60-0.82 (Additional file 1, Table S5). The frequencies of rs3110697 and rs924140 located in intron 3 and the promoter region of *IGFBP3 *and rs9658233 of *IGFBP1 *more closely resemble the frequencies of the HapMap YRI samples, suggesting African influence for these markers. Several genome wide association studies performed in European populations have replicated associations with SNPs in *IGF2BP2 *[44-47], implicating a role for the IGF pathway and T2D susceptibility.

We also note in the results that 2- and 3-SNP moving windows analyses were not required to capture ungenotyped YRI and CEU HapMap SNPs in *IL6, GCK1, IGFBP1 *and *IGFBP3*.

One limitation of this study is that all controls did not have measures of diabetes status or renal impairment. Serum glucose values were obtained for 42.9% (256 individuals) of the African American controls (mean 93.6 mg/dl). Five of these individuals had glucose levels greater than 126 mg/dl. Four of these measures were non-fasting and the fifth had unknown dietary status, suggesting the overall misclassification rate of controls is likely to be below 2%. While this has not impacted our ability to detect an association, this may have reduced our power to detect more subtle influences of additional variants, and underestimate effect sizes.

While adjustments for admixture reduced the strength for the observed associations with T2D-ESRD (Additional file 1, Table S5), associations with *IGFBP1 *rs9658233 and *IGFBP3 *(rs10255707, rs3110697, rs924140 and rs903889) remained nominally significant. Multiple comparisons were corrected by conservative Bonferroni method (P ≤ 0.00005) however, when assuming independence based on LD, one would consider a P-value ≤ 0.0007 as evidence for association, only SNP rs730497 remained significant (T2D age-adjusted *P *= 0.0006).

We estimate 65.7% power to detect an OR of 1.30 at a significance level of 0.05 in the case-control cohort [27].

We have confirmed linkage to early-onset T2D on chromosome 7p, and examined common variants across positional candidate genes *GCK1, IL6*, *IGFBPI *and *IGFBP3*. A variant of the *GCK1 *gene, rs730497, appears to play a role in susceptibility to earlier age of ESRD onset in African Americans. This may not represent the "true" casual variant but may be in LD with a functional SNP. Denser SNP genotyping and direct sequencing along with functional studies investigating intron 1 of the *GCK1 *gene are imperative to investigate the etiology of ESRD risk. It is plausible that multiple rare variants in this region of the gene may contribute to this phenotype. In addition, other candidates under the LOD-1 interval, includes the growth factor receptor-bound protein 10 (*GRB10*) [48,49] and IGF2 mRNA binding protein 3 (*IGF2BP3*), warrant investigation.

## Conclusion

Our findings support that intron 1 of the *GCK1 *play a role with ESRD risk. Further studies in other populations and functional studies may be warranted.

## Competing interests

The authors declare that they have no competing interests.

## Authors' contributions

TSL carried out the genotyping of additional polymorphic markers, prepared linkage data for analysis, performed association analysis, and drafted the manuscript. CDL developed software programs SNPGWA and QSNPGWA, and directed LL in conducting linkage analyses. KLK performed the admixture analysis. JCM assisted with statistical analysis. CJG assisted in preparing linkage data for analysis. SSR, BIF and DWB participated in the design of the study and participant recruitment. MMS conceived of the study, participated in its design and coordination and helped draft the manuscript. All authors read and approved the final manuscript.

## Pre-publication history

The pre-publication history for this paper can be accessed here:

http://www.biomedcentral.com/1471-2350/11/22/prepub

## Supplementary Material

Additional file 1 **Supplementary tables**. Supplemental tables S1-S6. Click here for file
