# Supplementary material for: Chromosome 7p linkage and association study for diabetes related traits and type 2 diabetes in an African-American population enriched for nephropathy
Source: BMC Med Genet. 2010 Feb 8;11:22. doi: 10.1186/1471-2350-11-22 (PMC2829011; doi:10.1186/1471-2350-11-22)
Supplement: Additional file 1 — Supplementary tables. Supplemental tables S1-S6. [file 1471-2350-11-22-S1.DOC]

**Supplementary Table 1 Single SNP association results for age of type 2 diabetes diagnosis**

| **Gene** | **Marker** | **1/1** | **1/2** | **2/2** | **Dominant P-value** | **Additive P-value** | **Recessive P-value** |
| --- | --- | --- | --- | --- | --- | --- | --- |
|  |  | **Mean ± SD (n)** | **Mean ± SD (n)** | **Mean ± SD (n)** |  |  |  |
| *IL-6* | rs12700386 | 41.5±11.3 (406) | 42.8±12.6 (155) | 38.6±10.3 (14) | 0.381 | 0.634 | 0.315 |
| *IL-6* | rs1800797 | 41.9±11.8 (508) | 41.9±10.9 (56) | 46.5±6.4 (2) | 0.867 | - | - |
| *IL-6* | rs1800796 | 41.9±11.4 (454) | 41.6±12.7 (118) | 38.0±2.8 (2) | 0.755 | - | - |
| *IL-6* | rs1800795 | 41.8±11.8 (512) | 42.1±10.7 (61) | 46.5±6.4 (2) | 0.745 | - | - |
| *IL-6* | rs2069830 | 41.9±11.6 (485) | 41.3±11.5 (81) | 50.0±16.9 (6) | 0.914 | - | - |
| *IL-6* | rs2069835 | 41.9±11.7 (468) | 41.2±11.3 (99) | 47.4±15.8 (8) | 0.776 | - | - |
| *IL-6* | rs1474347 | 41.4±11.9 (421) | 43.1±10.9 (140) | 40.6±11.0 (14) | 0.194 | 0.313 | 0.701 |
| *IL-6* | rs1524107 | 42.0±11.6 (469) | 40.9±12.0 (104) | 38.0±2.8 (2) | 0.386 | - | - |
| *IL-6* | rs2069840 | 41.9±11.7 (406) | 41.9±11.4 (155) | 36.8±12.0 (14) | 0.597 | 0.335 | 0.101 |
| *IL-6* | rs1554606 | 41.1±11.8 (255) | 42.3±11.7 (245) | 42.9±10.7 (68) | 0.167 | 0.162 | 0.416 |
| *IL-6* | rs2069842 | 41.9±11.7 (481) | 41.8±11.6 (89) | 34.7±7.5 (3) | 0.813 | - | - |
| *IL-6* | rs2069843 | 41.5±11.6 (414) | 43.3±11.9 (129) | 41.3±10.4 (9) | 0.154 | 0.210 | 0.866 |
| *IL-6* | rs2069860 | 41.8±11.7 (572) | 41.0±1.4 (2) | 0 (0) | 0.918 | - | - |
| *IL-6* | rs2069849 | 41.5±11.6 (419) | 42.7±11.7 (140) | 43.2±12.3 (16) | 0.258 | 0.261 | 0.642 |
| *IL-6* | rs2069861 | 41.8±11.6 (560) | 45.8±10.9 (14) | 0 (0) | 0.230 | - | - |
| *IL-6* | rs10242595 | 41.4±12.4 (165) | 42.2±11.3 (276) | 41.6±11.3 (134) | 0.591 | 0.839 | 0.819 |
| *IL-6* | rs11766273 | 41.8±11.7 (561) | 41.0±8.1 (14) | 0 (0) | 0.797 | - | - |
| *GCK1* | rs2908277 | 41.7±11.6 (311) | 42.1±11.9 (232) | 40.8±9.8 (32) | 0.769 | 0.952 | 0.625 |
| *GCK1* | rs2268576 | 41.9±11.9 (291) | 42.3±11.5 (232) | 39.2±9.9 (52) | 0.815 | 0.355 | 0.092 |
| *GCK1* | rs2268575 | 41.4±11.9 (443) | 43.8±10.3 (124) | 33.0±8.4 (6) | 0.102 | - | - |
| *GCK1* | rs2908296 | 41.6±11.0 (235) | 42.8±12.2 (277) | 38.0±10.3 (63) | 0.733 | 0.313 | **0.008** |
| *GCK1* | rs2971676 | 42.1±11.4 (328) | 41.8±11.8 (217) | 38.9±12.3 (30) | 0.512 | 0.296 | 0.182 |
| *GCK1* | rs2268572 | 41.5±11.9 (191) | 42.5±11.4 (269) | 40.8±11.7 (115) | 0.676 | 0.776 | 0.317 |
| *GCK1* | rs2971675 | 42.1±11.4 (338) | 41.6±11.9 (210) | 39.1±12.5 (27) | 0.441 | 0.281 | 0.235 |
| *GCK1* | rs758989 | 42.0±11.7 (394) | 41.7±11.4 (157) | 39.3±12.2 (24) | 0.570 | 0.405 | 0.308 |
| *GCK1* | rs2080033 | 42.2±11.6 (350) | 41.2±11.5 (191) | 41.4±12.6 (34) | 0.362 | 0.418 | 0.855 |
| *GCK1* | rs2971672 | 41.5±11.7 (208) | 42.2±12.1 (263) | 41.8±10.7 (90) | 0.551 | 0.708 | 0.937 |
| *GCK1* | rs12673242 | 41.3±11.38 (236) | 43.2±11.8 (258) | 39.0±11.2 (81) | 0.380 | 0.606 | **0.023** |
| *GCK1* | rs2908292 | 41.7±11.4 (399) | 42.3±12.2 (156) | 40.7±11.3 (20) | 0.668 | 0.827 | 0.664 |
| *GCK1* | rs2908290 | 41.3±11.6 (175) | 42.3±12.2 (269) | 41.5±10.5 (131) | 0.465 | 0.788 | 0.733 |
| *GCK1* | rs2284777 | 41.7±11.8 (497) | 42.8±10.8 (59) | 42.6±8.0 (11) | 0.473 | 0.507 | 0.815 |
| *GCK1* | rs2300586 | 41.4±11.6 (291) | 42.6±11.9 (244) | 39.5±10.3 (40) | 0.455 | 0.925 | 0.211 |
| *GCK1* | rs2300584 | 41.7±11.6 (438) | 42.4±11.7 (125) | 38.0±12.1 (12) | 0.788 | 0.934 | 0.271 |
| *GCK1* | rs758988 | 42.2±11.5 (441) | 40.8±12.3 (123) | 39.0±10.0 (11) | 0.199 | 0.175 | 0.463 |
| *GCK1* | rs758985 | 41.8±11.6 (486) | 42.4±11.4 (80) | 37.6±12.8 (9) | 0.997 | - | - |
| *GCK1* | rs1990458 | 41.0±11.2 (349) | 43.5±12.0 (196) | 39.9±12.5 (30) | 0.057 | 0.226 | 0.369 |
| *GCK1* | rs741038 | 42.0±12.0 (374) | 41.6±10.8 (155) | 41.3±11.4 (39) | 0.682 | 0.663 | 0.771 |
| *GCK1* | rs730497 | 42.3±11.3 (367) | 41.3±12.4 (180) | 38.3±9.5 (25) | 0.178 | 0.093 | 0.126 |
| *GCK1* | rs2908289 | 41.9±11.3 (315) | 42.3±12.5 (211) | 38.7±9.5 (49) | 0.719 | 0.259 | 0.051 |
| *GCK1* | rs6952751 | 41.6±11.7 (500) | 43.2±11.3 (72) | 38.7±6.7 (3) | 0.367 | - | - |
| *GCK1* | rs735670 | 41.7±11.7 (524) | 42.2±11.3 (46) | 50.5±6.4 (5) | 0.496 | - | - |
| *IGFBP1* | rs4724445 | 41.6±11.4 (502) | 43.3±13.7 (63) | 55.0±7.1 (2) | 0.180 | - | - |
| *IGFBP1* | rs3763497 | 42.2±11.6 (394) | 40.8±11.5 (161) | 44.8±13.8 (12) | 0.311 | 0.514 | 0.377 |
| *IGFBP1* | rs1065780 | 41.7±11.1 (194) | 41.7±12.2 (279) | 42.3±11.3 (94) | 0.884 | 0.729 | 0.648 |
| *IGFBP1* | rs3828998 | 41.6±11.2 (188) | 41.9±12.1 (279) | 42.2±11.2 (100) | 0.713 | 0.671 | 0.748 |
| *IGFBP1* | rs3793344 | 41.9±11.3 (197) | 41.6±12.1 (274) | 42.3±11.3 (96) | 0.890 | 0.884 | 0.655 |
| *IGFBP1* | rs4619 | 41.8±11.1 (174) | 41.6±11.9 (282) | 42.4±11.9 (111) | 0.993 | 0.724 | 0.538 |
| *IGFBP1* | rs7454 | 41.7±11.7 (546) | 47.2±10.3 (18) | 32.0±0.0 (1) | 0.093 | - | - |
| *IGFBP1* | rs1908750 | 41.9±11.6 (531) | 39.4±11.9 (35) | 42.0±0.0 (1) | 0.237 | - | - |
| *IGFBP1* | rs9658231 | 41.9±11.4 (472) | 41.4±12.8 (90) | 34.8±6.9 (4) | 0.558 | - | - |
| *IGFBP1* | rs9658233 | 41.3±11.2 (383) | 42.8±12.3 (172) | 43.0±14.5 (11) | 0.142 | 0.153 | 0.735 |
| *IGFBP1* | rs9658238 | 41.8±11.8 (450) | 41.7±10.9 (113) | 49.0±13.1 (4) | 0.840 | - | - |
| *IGFBP1* | rs9658239 | 41.9±11.7 (527) | 40.5±11.1 (30) | 64.0±0.0 (1) | 0.783 | - | - |
| *IGFBP1* | rs1908751 | 41.6±10.9 (254) | 42.6±12.5 (243) | 39.5±11.7 (60) | 0.719 | 0.640 | 0.114 |
| *IGFBP1* | rs13442660 | 41.9±11.1 (176) | 41.6±11.9 (284) | 42.2±11.7 (107) | 0.929 | 0.890 | 0.724 |
| *IGFBP1* | rs1496495 | 41.8±11.8 (466) | 41.9±11.2 (79) | 39.4±10.6 (12) | 0.883 | 0.718 | 0.479 |
| *IGFBP1* | rs13441765 | 41.4±11 (307) | 42.7±12.3 (221) | 40.6±12.9 (38) | 0.315 | 0.591 | 0.506 |
| *IGFBP3* | rs12671484 | 41.7±11.6 (527) | 42.7±11.9 (37) | 64.0±0.0 (1) | 0.462 | - | - |
| *IGFBP3* | rs2270628 | 41.7±11.2 (217) | 41.5±12.1 (280) | 43.4±11.4 (70) | 0.834 | 0.468 | 0.248 |
| *IGFBP3* | rs13223993 | 41.3±10.9 (172) | 42.2±11.8 (276) | 41.7±12.4 (119) | 0.536 | 0.741 | 0.903 |
| *IGFBP3* | rs6670 | 41.9±11.9 (447) | 41.2±10.6 (114) | 41.2±11.7 (6) | 0.501 | - | - |
| *IGFBP3* | rs10255707 | 41.7±11.7 (511) | 44.5±11.6 (33) | 42.0±0.0 (2) | 0.216 | - | - |
| *IGFBP3* | rs3110697 | 41.2±11.1 (234) | 42.2±12.2 (270) | 42.3±11.5 (63) | 0.359 | 0.394 | 0.730 |
| *IGFBP3* | rs6953668 | 41.8±11.2 (494) | 42.1±14.4 (71) | 0 (0) | 0.836 | - | - |
| *IGFBP3* | rs2453837 | 41.8±11.7 (531) | 42.2±11.7 (33) | 50.0±0.0 (1) | 0.747 | - | - |
| *IGFBP3* | rs924140 | 41.9±11.5 (200) | 41.7±11.9 (276) | 41.3±11.2 (80) | 0.730 | 0.664 | 0.717 |
| *IGFBP3* | rs903889 | 41.8±11.4 (475) | 41.8±13.0 (91) | 60.0±0.0 (1) | 0.834 | - | - |
| *IGFBP3* | rs2453836 | 41.8±11.8 (356) | 41.9±11.5 (189) | 41.1±10.1 (21) | 0.994 | 0.918 | 0.775 |

1 denotes the major allele and 2 the minor allele. **Bold**: P-values <0.05

**Supplementary Table 2 Single SNP association results for age at end-stage renal disease** onset

| **Gene** | **Marker** | **1/1** | **1/2** | **2/2** | **Dominant P-value** | **Additive P-value** | **Recessive P-value** |
| --- | --- | --- | --- | --- | --- | --- | --- |
|  |  | **Mean ± SD (n)** | **Mean ± SD (n)** | **Mean ± SD (n)** |  |  |  |
| *IL-6* | rs12700386 | 59.1±10.4 (406) | 59.2±10.7 (155) | 57.7±10.7 (14) | 0.998 | 0.885 | 0.626 |
| *IL-6* | rs1800797 | 59.1±10.5 (508) | 58.3±10.4 (56) | 60.0±5.7 (2) | 0.619 | - | - |
| *IL-6* | rs1800796 | 58.7±10.5 (454) | 60.5±10.7 (118) | 56.5±2.1 (2) | 0.110 | - | - |
| *IL-6* | rs1800795 | 59.2±10.5 (512) | 58.3±10.3 (61) | 60.0±5.7 (2) | 0.553 | - | - |
| *IL-6* | rs2069830 | 59.2±10.5 (485) | 58.9±10.3 (81) | 60.2±9.5 (6) | 0.878 | - | - |
| *IL-6* | rs2069835 | 59.2±10.6 (468) | 58.4±10.1 (99) | 58.4±8.8 (8) | 0.498 | - | - |
| *IL-6* | rs1474347 | 59.1±10.4 (421) | 59.2±10.8 (140) | 57.4±10.5 (14) | 0.915 | 0.777 | 0.538 |
| *IL-6* | rs1524107 | 58.9±10.5 (469) | 59.9±10.5 (104) | 56.5±2.1 (2) | 0.394 | - | - |
| *IL-6* | rs2069840 | 59.1±10.2 (406) | 58.8±11.2 (155) | 60.1±11.8 (14) | 0.848 | 0.959 | 0.697 |
| *IL-6* | rs1554606 | 59.2±10.5 (255) | 59.5±10.3 (245) | 56.9±10.9 (68) | 0.726 | 0.260 | 0.070 |
| *IL-6* | rs2069842 | 59.2±10.3 (481) | 58.7±11.6 (89) | 55.7±4.5 (3) | 0.653 | - | - |
| *IL-6* | rs2069843 | 58.9±10.5 (414) | 59.9±10.8 (129) | 51.7±6.9 (9) | 0.659 | - | **-** |
| *IL-6* | rs2069860 | 59.1±10.5 (572) | 51.5±4.9 (2) | 0 (0) | 0.306 | - | - |
| *IL-6* | rs2069849 | 59.0±10.5 (419) | 59.8±10.4 (140) | 53.7±10.4 (16) | 0.896 | 0.581 | **0.040** |
| *IL-6* | rs2069861 | 59.1±10.5 (560) | 59.4±8.9 (14) | 0 (0) | 0.901 | - | - |
| *IL-6* | rs10242595 | 59.3±9.6 (165) | 59.2±10.9 (276) | 58.5±10.7 (134) | 0.738 | 0.510 | 0.445 |
| *IL-6* | rs11766273 | 59.1±10.6 (561) | 56.6±7.8 (14) | 0 (0) | 0.395 | - | - |
| *GCK1* | rs2908277 | 59.5±10.5 (311) | 58.2±10.7 (232) | 58.9±9.6 (32) | 0.198 | 0.285 | 0.999 |
| *GCK1* | rs2268576 | 59.4±10.4 (291) | 58.9±10.5 (232) | 57.2±11.4 (52) | 0.378 | 0.226 | 0.226 |
| *GCK1* | rs2268575 | 58.7±10.7 (443) | 59.9±9.9 (124) | 59.0±4.9 (6) | 0.336 | - | - |
| *GCK1* | rs2908296 | 59.2±10.0 (235) | 59.4±11.2 (277) | 56.0±8.7 (63) | 0.632 | 0.157 | **0.027** |
| *GCK1* | rs2971676 | 59.4±10.4 (328) | 59.0±10.8 (217) | 54.4±8.8 (30) | 0.328 | 0.092 | **0.020** |
| *GCK1* | rs2268572 | 58.9±10.1 (191) | 59.2±11.0 (269) | 58.4±10.1 (115) | 0.934 | 0.772 | 0.539 |
| *GCK1* | rs2971675 | 59.5±10.5 (338) | 58.7±10.8 (210) | 54.5±8.4 (27) | 0.200 | 0.065 | **0.033** |
| *GCK1* | rs758989 | 59.3±10.4 (394) | 58.5±11.0 (157) | 56.6±9.2 (24) | 0.315 | 0.225 | 0.288 |
| *GCK1* | rs2080033 | 59.3±10.3 (350) | 58.6±11.0 (191) | 57.6±10.1 (34) | 0.381 | 0.323 | 0.472 |
| *GCK1* | rs2971672 | 58.3±10.6 (208) | 59.8±10.4 (263) | 58.0±10.9 (90) | 0.254 | 0.724 | 0.402 |
| *GCK1* | rs12673242 | 58.6±9.9 (236) | 59.7±11.0 (258) | 57.7±10.7 (81) | 0.491 | 0.935 | 0.253 |
| *GCK1* | rs2908292 | 58.9±10.6 (399) | 59.2±10.3 (156) | 57.5±1.3 (20) | 0.892 | 0.932 | 0.548 |
| *GCK1* | rs2908290 | 58.5±10.9 (175) | 59.5±10.6 (269) | 58.5±9.9 (131) | 0.480 | 0.911 | 0.560 |
| *GCK1* | rs2284777 | 58.9±10.7 (497) | 59.4±9.7 (59) | 57.5±4.3 (11) | 0.932 | 0.927 | 0.650 |
| *GCK1* | rs2300586 | 58.8±10.2 (291) | 59.2±11.0 (244) | 58.6±10.1 (40) | 0.695 | 0.828 | 0.808 |
| *GCK1* | rs2300584 | 58.9±10.7 (438) | 59.1±9.8 (125) | 57.0±11.9 (12) | 0.931 | 0.805 | 0.551 |
| *GCK1* | rs758988 | 59.4±10.3 (441) | 57.6±11.4 (123) | 56.3±9.4 (11) | 0.084 | 0.078 | 0.450 |
| *GCK1* | rs758985 | 59.1±10.5 (486) | 58.9±9.6 (80) | 52.1±16.4 (9) | 0.432 | - | **-** |
| *GCK1* | rs1990458 | 58.7±10.2 (349) | 59.5±11.1 (196) | 58.6±1.8 (30) | 0.449 | 0.589 | 0.844 |
| *GCK1* | rs741038 | 59.3±10.6 (374) | 58.3±9.3 (155) | 57.8±12.9 (39) | 0.229 | 0.226 | 0.491 |
| *GCK1* | rs730497 | 58.9±10.4 (367) | 60.0±10.6 (180) | 51.5±8.6 (25) | 0.989 | 0.206 | **0.0004** |
| *GCK1* | rs2908289 | 58.4±10.5 (315) | 60.6±10.7 (211) | 55.1±8.8 (49) | 0.223 | 0.827 | **0.008** |
| *GCK1* | rs6952751 | 58.6±10.7 (500) | 61.1±9.6 (72) | 58.7±2.3 (3) | 0.081 | - | - |
| *GCK1* | rs735670 | 58.8±10.6 (524) | 59.9±10.4 (46) | 66.5±7.5 (5) | 0.291 | - | - |
| *IGFBP1* | rs4724445 | 59.0±10.4 (502) | 59.5±11.5 (63) | 59.5±3.5 (2) | 0.742 | - | - |
| *IGFBP1* | rs3763497 | 59.2±10.4 (394) | 58.6±11.0 (161) | 61.0±9.6 (12) | 0.653 | 0.824 | 0.527 |
| *IGFBP1* | rs1065780 | 58.9±10.3 (194) | 59.2±10.9 (279) | 59.2±9.8 (94) | 0.714 | 0.772 | 0.946 |
| *IGFBP1* | rs3828998 | 58.9±10.1 (188) | 59.3±11.0 (279) | 58.8±9.9 (100) | 0.747 | 0.953 | 0.772 |
| *IGFBP1* | rs3793344 | 58.9±10.3 (197) | 59.2±10.9 (274) | 59.0±9.7 (96) | 0.850 | 0.938 | 0.923 |
| *IGFBP1* | rs4619 | 58.9±10.5 (174) | 58.9±10.9 (282) | 59.7±9.7 (111) | 0.818 | 0.600 | 0.510 |
| *IGFBP1* | rs7454 | 59.1±10.6 (546) | 60.8±7.9 (18) | 41.0±0.0 (1) | 0.795 | - | - |
| *IGFBP1* | rs1908750 | 59.1±10.6 (531) | 58.8±8.9 (35) | 64.0±0.0 (1) | 0.942 | - | - |
| *IGFBP1* | rs9658231 | 59.0±10.6 (472) | 58.9±10.1 (90) | 63.3±2.6 (4) | 0.928 | - | - |
| *IGFBP1* | rs9658233 | 58.7±10.5 (383) | 59.7±10.4 (172) | 61.7±10.7 (11) | 0.255 | 0.206 | 0.396 |
| *IGFBP1* | rs9658238 | 59.2±10.4 (450) | 58.7±10.9 (113) | 57.5±12.4 (4) | 0.640 | - | - |
| *IGFBP1* | rs9658239 | 59.1±10.6 (527) | 59.4±10.5 (30) | 71.0±0.0 (1) | 0.699 | - | - |
| *IGFBP1* | rs1908751 | 58.9±10.1 (254) | 59.4±10.7 (243) | 58.0±12.0 (60) | 0.863 | 0.809 | 0.428 |
| *IGFBP1* | rs13442660 | 58.9±10.5 (176) | 59.1±10.9 (284) | 59.5±9.7 (107) | 0.817 | 0.708 | 0.695 |
| *IGFBP1* | rs1496495 | 58.9±10.5 (466) | 59.7±10.4 (79) | 55.7±10.9 (12) | 0.832 | 0.851 | 0.268 |
| *IGFBP1* | rs13441765 | 58.6±10.7 (307) | 59.8±10.3 (221) | 59.6±10.3 (38) | 0.166 | 0.219 | 0.766 |
| *IGFBP3* | rs12671484 | 58.9±10.5 (527) | 60.3±10.9 (37) | 71.0±0.0 (1) | 0.340 | - | - |
| *IGFBP3* | rs2270628 | 59.1±10.1 (217) | 58.8±11.1 (280) | 60.4±9.5 (70) | 0.945 | 0.570 | 0.295 |
| *IGFBP3* | rs13223993 | 59.4±10.3 (172) | 58.8±10.7 (276) | 59.3±10.5 (119) | 0.706 | 0.922 | 0.798 |
| *IGFBP3* | rs6670 | 58.9±10.7 (447) | 59.8±9.8 (114) | 62.3±5.9 (6) | 0.322 | - | - |
| *IGFBP3* | rs10255707 | 59.1±10.6 (511) | 59.8±11.7 (33) | 57.5±2.1 (2) | 0.754 | - | - |
| *IGFBP3* | rs3110697 | 58.7±10.8 (234) | 59.3±10.6 (270) | 59.9±9.4 (63) | 0.421 | 0.369 | 0.536 |
| *IGFBP3* | rs6953668 | 59.3±10.4 (494) | 57.9±11.5 (71) | 0 (0) | 0.316 | - | - |
| *IGFBP3* | rs2453837 | 58.9±10.5 (531) | 60.8±10.0 (33) | 72.0±0.0 (1) | 0.233 | 0.176 | 0.217 |
| *IGFBP3* | rs924140 | 59.2±11.1 (200) | 58.9±10.1 (276) | 59.8±10.6 (80) | 0.935 | 0.836 | 0.608 |
| *IGFBP3* | rs903889 | 59.2±10.4 (475) | 58.3±11.3 (91) | 62.0±0.0 (1) | 0.473 | - | - |
| *IGFBP3* | rs2453836 | 58.6±10.9 (356) | 60.1±9.7 (189) | 58.3±8.8 (21) | 0.159 | 0.266 | 0.737 |

1 denotes the major allele and 2 the minor allele. **Bold**: P-values <0.05.

**Supplementary Table 3 Single SNP association results for duration of type 2 diabetes to onset of end stage renal disease**

| **Gene** | **Marker** | **1/1**  **Mean±SD (n)** | **1/2**  **Mean±SD (n)** | **2/2**  **Mean±SD (n)** | **Dominant P-value** | **Additive P-value** | **Recessive P-value** |
| --- | --- | --- | --- | --- | --- | --- | --- |
| *IL-6* | rs12700386 | 19.8±13.8 (406) | 18.6±14.4 (155) | 21.8±16.9 (14) | 0.479 | 0.662 | 0.538 |
| *IL-6* | rs1800797 | 19.7±14.2 (508) | 18.3±14.2 (56) | 13.5±0.71 (2) | 0.433 | - | - |
| *IL-6* | rs1800796 | 19.5±14.6 (454) | 19.7±12.0 (118) | 18.5±5.0 (2) | 0.932 | - | - |
| *IL-6* | rs1800795 | 19.8±14.1 (512) | 18.5±13.8 (61) | 13.5±0.7 (2) | 0.323 | - | - |
| *IL-6* | rs2069830 | 19.4±13.8 (485) | 20.8±14.7 (81) | 26.8±26.6 (6) | 0.496 | - | - |
| *IL-6* | rs2069835 | 19.5±13.9 (468) | 18.8±13.4 (99) | 28.7±24.2 (8) | 0.997 | - | - |
| *IL-6* | rs1474347 | 19.9±14.1 (421) | 18.7±14.3 (140) | 16.7±11.5 (14) | 0.291 | 0.246 | 0.442 |
| *IL-6* | rs1524107 | 19.5±14.5 (469) | 19.8±12.2 (104) | 18.5±5.0 (2) | 0.834 | - | - |
| *IL-6* | rs2069840 | 19.5±14.1 (406) | 19.3±14.4 (155) | 23.3±9.1 (14) | 0.858 | 0.641 | 0.308 |
| *IL-6* | rs1554606 | 20.2±13.7 (255) | 19.7±14.7 (245) | 15.8±13.0 (68) | 0.247 | 0.052 | **0.022** |
| *IL-6* | rs2069842 | 19.5±13.9 (481) | 19.3±14.6 (89) | 21.0±3.0 (3) | 0.935 | - | - |
| *IL-6* | rs2069843 | 19.7±14.1 (414) | 18.3±13.8 (129) | 15.0±15.0 (9) | 0.266 | - | - |
| *IL-6* | rs2069860 | 19.6±14.1 (572) | 10.5±6.4 (2) | 0 (0) | 0.362 | - | - |
| *IL-6* | rs2069849 | 19.7±14.1 (419) | 19.7±14.8 (140) | 13.2±13.3 (16) | 0.572 | 0.282 | 0.068 |
| *IL-6* | rs2069861 | 19.5±14.1 (560) | 20.1±16.2 (14) | 0 (0) | 0.878 | - | - |
| *IL-6* | rs10242595 | 20.4±13.4 (165) | 19.3±14.9 (276) | 19.1±12.9 (134) | 0.374 | 0.418 | 0.670 |
| *IL-6* | rs11766273 | 19.7±14.2 (561) | 14.5±8.3 (14) | 0 (0) | 0.172 | - | - |
| *GCK1* | rs2908277 | 19.5±13.5 (311) | 19.3±14.7 (232) | 21.6±14.8 (32) | 0.960 | 0.704 | 0.380 |
| *GCK1* | rs2268576 | 19.7±14.3 (291) | 19.3±14.2 (232) | 19.4±12.3 (52) | 0.742 | 0.778 | 0.950 |
| *GCK1* | rs2268575 | 19.6±13.9 (443) | 18.4±14.0 (124) | 34.5±21.8 (6) | 0.719 | - | **-** |
| *GCK1* | rs2908296 | 20.1±15.2 (235) | 18.8±13.2 (277) | 20.4±13.1 (63) | 0.424 | 0.726 | 0.607 |
| *GCK1* | rs2971676 | 20.1±14.9 (328) | 18.8±12.5 (217) | 19.0±15.0 (30) | 0.318 | 0.361 | 0.820 |
| *GCK1* | rs2268572 | 20.2±13.6 (191) | 19.1±14.3 (269) | 19.6±14.3 (115) | 0.459 | 0.642 | 0.971 |
| *GCK1* | rs2971675 | 20.1±14.7 (338) | 18.8±12.7 (210) | 19.3±15.5 (27) | 0.326 | 0.395 | 0.940 |
| *GCK1* | rs758989 | 19.5±13.6 (394) | 19.4±14.7 (157) | 22.4±16.5 (24) | 0.824 | 0.616 | 0.379 |
| *GCK1* | rs2080033 | 19.3±13.9 (350) | 19.7±14.1 (191) | 20.5±15.3 (34) | 0.678 | 0.620 | 0.681 |
| *GCK1* | rs2971672 | 19.1±13.3 (208) | 19.2±13.5 (263) | 20.0±16.1 (90) | 0.795 | 0.660 | 0.621 |
| *GCK1* | rs12673242 | 20.2±15.2 (236) | 18.3±12.8 (258) | 21.3±14.2 (81) | 0.325 | 0.922 | 0.228 |
| *GCK1* | rs2908292 | 20.3±14.5 (399) | 18.5±12.8 (156) | 18.6±15.1 (20) | 0.249 | 0.279 | 0.768 |
| *GCK1* | rs2908290 | 18.8±12.8 (175) | 19.5±13.9 (269) | 20.4±15.7 (131) | 0.439 | 0.340 | 0.421 |
| *GCK1* | rs2284777 | 19.5±13.8 (497) | 19.8±15.4 (59) | 16.5±9.2 (11) | 0.930 | 0.761 | 0.493 |
| *GCK1* | rs2300586 | 19.8±14.7 (291) | 18.7±13.5 (244) | 22.6±15.0 (40) | 0.636 | 0.845 | 0.157 |
| *GCK1* | rs2300584 | 19.7±14.2 (438) | 18.7±13.2 (125) | 22.1±18.1 (12) | 0.588 | 0.764 | 0.534 |
| *GCK1* | rs758988 | 19.8±14.0 (441) | 18.1±13.5 (123) | 25.1±20.4 (11) | 0.453 | 0.781 | 0.181 |
| *GCK1* | rs758985 | 19.3±13.5 (486) | 21.2±17.3 (80) | 14.5±9.2 (9) | 0.478 | - | - |
| *GCK1* | rs1990458 | 20.2±14.4 (349) | 18.4±13.8 (196) | 18.6±11.2 (30) | 0.154 | 0.191 | 0.708 |
| *GCK1* | rs741038 | 19.3±13.4 (374) | 18.8±13.3 (155) | 22.6±19.6 (39) | 0.841 | 0.444 | 0.138 |
| *GCK1* | rs730497 | 19.4±15.0 (367) | 20.5±12.3 (180) | 13.3±7.1 (25) | 0.797 | 0.588 | 0.031 |
| *GCK1* | rs2908289 | 19.2±14.8 (315) | 20.6±13.8 (211) | 16.8±8.9 (49) | 0.541 | 0.908 | 0.173 |
| *GCK1* | rs6952751 | 19.4±13.9 (500) | 20.6±15.3 (72) | 20.0±6.6 (3) | 0.473 | - | - |
| *GCK1* | rs735670 | 19.4±13.6 (524) | 20.3±17.8 (46) | 28.2±27.5 (5) | 0.404 | - | - |
| *IGFBP1* | rs4724445 | 20.1±14.2 (502) | 17.8±12.9 (63) | 19.7±14.1 (2) | 0.168 | - | - |
| *IGFBP1* | rs3763497 | 19.9±14.2 (394) | 19.4±13.9 (161) | 19.7±14.1 (12) | 0.602 | 0.477 | 0.392 |
| *IGFBP1* | rs1065780 | 19.1±13.5 (194) | 20.6±15.8 (279) | 20.4±14.4 (94) | 0.546 | 0.795 | 0.213 |
| *IGFBP1* | rs3828998 | 19.3±13.6 (188) | 20.6±15.1 (279) | 20.1±14.5 (100) | 0.657 | 0.602 | 0.135 |
| *IGFBP1* | rs3793344 | 19.8±13.5 (197) | 20.7±15.1 (274) | 20.5±14.4 (96) | 0.454 | 0.872 | 0.213 |
| *IGFBP1* | rs4619 | 18.7±13.5 (174) | 20.5±14.9 (282) | 19.8±14.3 (111) | 0.317 | 0.733 | 0.581 |
| *IGFBP1* | rs7454 | 19.8±14.1 (546) | 16.1±14.7 (18) | 19.7±14.1 (1) | 0.221 | - | - |
| *IGFBP1* | rs1908750 | 19.4±13.9 (531) | 23.5±16.2 (35) | 19.7±14.1 (1) | 0.104 | - | - |
| *IGFBP1* | rs9658231 | 19.6±14.5 (472) | 19.5±12.4 (90) | 19.6±14.1 (4) | 0.878 | - | - |
| *IGFBP1* | rs9658233 | 19.5±13.9 (383) | 19.9±14.3 (172) | 19.6±14.0 (11) | 0.680 | 0.574 | 0.489 |
| *IGFBP1* | rs9658238 | 19.8±13.9 (450) | 19.5±14.9 (113) | 19.7±14.1 (4) | 0.648 | - | - |
| *IGFBP1* | rs9658239 | 19.5±13.8 (527) | 21.6±17.9 (30) | 19.6±14.0 (1) | 0.521 | - | - |
| *IGFBP1* | rs1908751 | 19.5±13.4 (254) | 20.2±14.6 (243) | 19.6±14.0 (60) | 0.310 | 0.347 | 0.693 |
| *IGFBP1* | rs13442660 | 18.7±13.4 (176) | 20.5±14.8 (284) | 19.8±14.3 (107) | 0.266 | 0.638 | 0.639 |
| *IGFBP1* | rs1496495 | 19.3±13.7 (466) | 21.7±17.1 (79) | 19.7±14.2 (12) | 0.317 | 0.576 | 0.399 |
| *IGFBP1* | rs13441765 | 19.3±14.2 (307) | 19.9±14.2 (221) | 19.6±14.2 (38) | 0.522 | 0.436 | 0.512 |
| *IGFBP3* | rs12671484 | 19.6±13.9 (527) | 20.5±16.9 (37) | 19.7±14.1 (1) | 0.987 | - | - |
| *IGFBP3* | rs2270628 | 19.3±13.6 (217) | 20.2±14.7 (280) | 19.8±14.2 (70) | 0.687 | 0.966 | 0.498 |
| *IGFBP3* | rs13223993 | 19.4±13.6 (172) | 19.9±15.2 (276) | 19.7±14.5 (119) | 0.774 | 0.956 | 0.820 |
| *IGFBP3* | rs6670 | 19.7±14.6 (447) | 19.2±11.7 (114) | 19.6±14.0 (6) | 0.972 | - | - |
| *IGFBP3* | rs10255707 | 19.8±14.0 (511) | 18.9±15.9 (33) | 19.7±14.1 (2) | 0.435 | - | - |
| *IGFBP3* | rs3110697 | 21.3±15.7 (234) | 18.5±12.7 (270) | 19.8±14.2 (63) | **0.027** | 0.055 | 0.586 |
| *IGFBP3* | rs6953668 | 20.1±14.5 (494) | 16.8±11.2 (71) | 0 (0) | 0.086 | - | - |
| *IGFBP3* | rs2453837 | 19.6±14.2 (531) | 19.8±13.2 (33) | 19.6±14.1 (1) | 0.909 | - | - |
| *IGFBP3* | rs924140 | 20.9±15.5 (200) | 18.7±13.2 (276) | 19.6±14.2 (80) | 0.103 | 0.259 | 0.953 |
| *IGFBP3* | rs903889 | 20.8±14.3 (475) | 17.9±12.8 (91) | 19.7±14.1 (1) | 0.159 | - | - |
| *IGFBP3* | rs2453836 | 20.2±15.0 (356) | 19.1±12.6 (189) | 19.7±14.2 (21) | 0.507 | 0.575 | 0.966 |

1 denotes the major allele and 2 the minor allele. **Bold**: P-values <0.05.

Supplementary Table 4 Single SNP association results for body mass index (BMI)

| **Gene** | **Marker** | **1/1**  **Mean±SD (n)** | **1/2**  **Mean±SD (n)** | **2/2**  **Mean±SD (n)** | **Dominant P-value** | **Additive P-value** | **Recessive P-value** |
| --- | --- | --- | --- | --- | --- | --- | --- |
| *IL-6* | rs12700386 | 29.8±6.9 (406) | 29.3±6.9 (155) | 29.9±8.2 (14) | 0.482 | 0.569 | 0.869 |
| *IL-6* | rs1800797 | 29.6±7.0 (508) | 30.8±6.4 (56) | 27.4±2.2 (2) | 0.271 | - | - |
| *IL-6* | rs1800796 | 29.8±7.0 (454) | 29.1±6.6 (118) | 25.7±1.9 (2) | 0.291 | - | - |
| *IL-6* | rs1800795 | 29.6±7.0 (512) | 30.5±6.4 (61) | 27.3±2.2 (2) | 0.394 | - | - |
| *IL-6* | rs2069830 | 29.5±6.8 (485) | 30.2±7.5 (81) | 31.4±8.1 (6) | 0.361 | - | - |
| *IL-6* | rs2069835 | 29.6±6.8 (468) | 29.7±7.7 (99) | 31.6±7.0 (8) | 0.737 | - | - |
| *IL-6* | rs1474347 | 29.6±6.9 (421) | 29.5±7.0 (140) | 31.1±7.9 (14) | 0.973 | 0.832 | 0.431 |
| *IL-6* | rs1524107 | 29.7±7.0 (469) | 29.4±6.5 (104) | 25.7±1.9 (2) | 0.568 | - | - |
| *IL-6* | rs2069840 | 29.7±7.7 (406) | 29.7±7.6 (155) | 27.1±5.7 (14) | 0.761 | 0.504 | 0.175 |
| *IL-6* | rs1554606 | 29.8±7.3 (255) | 29.2±6.8 (245) | 30.7±6.4 (68) | 0.630 | 0.780 | 0.189 |
| *IL-6* | rs2069842 | 29.8±6.9 (481) | 29.2±7.2 (89) | 24.4±3.5 (3) | 0.366 | - | - |
| *IL-6* | rs2069843 | 29.6±7.1 (414) | 29.4±6.5 (129) | 33.8±6.2 (9) | 0.932 | - | - |
| *IL-6* | rs2069860 | 29.6±6.9 (572) | 28.7±3.7 (2) | 0 (0) | 0.855 | - | - |
| *IL-6* | rs2069849 | 29.7±7.1 (419) | 29.3±6.3 (140) | 31.4±7.4 (16) | 0.844 | 0.881 | 0.309 |
| *IL-6* | rs2069861 | 29.6±6.9 (560) | 30.6±7.6 (14) | 0 (0) | 0.612 | - | - |
| *IL-6* | rs10242595 | 30.1±6.9 (165) | 29.1±6.9 (276) | 30.2±6.9 (134) | 0.359 | 0.989 | 0.319 |
| *IL-6* | rs11766273 | 29.7±6.9 (561) | 28.5±4.9 (14) | 0 (0) | 0.549 | - | - |
| *GCK1* | rs2908277 | 29.0±6.7 (311) | 30.4±7.2 (232) | 28.6±6.1 (32) | 0.051 | 0.187 | 0.445 |
| *GCK1* | rs2268576 | 29.8±7.1 (291) | 29.3±6.9 (232) | 29.3±5.6 (52) | 0.364 | 0.403 | 0.748 |
| *GCK1* | rs2268575 | 29.5±6.9 (443) | 29.9±6.9 (124) | 31.3±6.5 (6) | 0.455 | - | - |
| *GCK1* | rs2908296 | 30.1±6.9 (235) | 28.8±6.8 (277) | 30.5±7.2 (63) | 0.099 | 0.504 | 0.238 |
| *GCK1* | rs2971676 | 29.6±7.0 (328) | 29.3±6.7 (217) | 31.7±7.3 (30) | 0.992 | 0.513 | 0.079 |
| *GCK1* | rs2268572 | 29.8±7.8 (191) | 29.3±6.5 (269) | 29.7±6.4 (115) | 0.487 | 0.717 | 0.872 |
| *GCK1* | rs2971675 | 29.5±6.9 (338) | 29.4±6.9 (210) | 31.7±7.0 (27) | 0.773 | 0.407 | 0.108 |
| *GCK1* | rs758989 | 29.6±7.2 (394) | 29.3±6.2 (157) | 30.1±6.8 (24) | 0.728 | 0.884 | 0.696 |
| *GCK1* | rs2080033 | 29.9±7.2 (350) | 28.9±6.6 (191) | 29.9±6.4 (34) | 0.134 | 0.275 | 0.751 |
| *GCK1* | rs2971672 | 29.8±7.1 (208) | 29.1±6.6 (263) | 30.7±7.6 (90) | 0.643 | 0.594 | 0.105 |
| *GCK1* | rs12673242 | 30.1±7.2 (236) | 28.8±6.5 (258) | 30.4±7.2 (81) | 0.105 | 0.562 | 0.257 |
| *GCK1* | rs2908292 | 29.7±7.1 (399) | 29.1±6.3 (156) | 30.5±8.0 (20) | 0.443 | 0.653 | 0.559 |
| *GCK1* | rs2908290 | 29.5±6.8 (175) | 29.2±6.8 (269) | 30.5±7.4 (131) | 0.919 | 0.306 | 0.097 |
| *GCK1* | rs2284777 | 29.5±7.1 (497) | 30.3±6.6 (59) | 29.7±3.7 (11) | 0.418 | 0.487 | 0.921 |
| *GCK1* | rs2300586 | 29.7±7.0 (291) | 29.2±6.8 (244) | 30.5±7.3 (40) | 0.563 | 0.907 | 0.396 |
| *GCK1* | rs2300584 | 29.8±7.1 (438) | 28.9±6.4 (125) | 28.4±4.6 (12) | 0.190 | 0.182 | 0.540 |
| *GCK1* | rs758988 | 29.4±7.0 (441) | 30.2±6.7 (123) | 27.4±6.3 (11) | 0.461 | 0.724 | 0.302 |
| *GCK1* | rs758985 | 29.7±7.1 (486) | 28.9±5.8 (80) | 27.1±8.2 (9) | 0.259 | - | - |
| *GCK1* | rs1990458 | 29.6±7.0 (349) | 29.7±6.8 (196) | 28.7±7.0 (30) | 0.987 | 0.792 | 0.464 |
| *GCK1* | rs741038 | 29.5±7.0 (374) | 29.8±6.6 (155) | 28.6±7.4 (39) | 0.974 | 0.747 | 0.398 |
| *GCK1* | rs730497 | 29.9±7.1 (367) | 28.8±6.6 (180) | 30.8±7.1 (25) | 0.168 | 0.402 | 0.372 |
| *GCK1* | rs2908289 | 29.7±6.6 (315) | 29.3±7.4 (211) | 30.1±7.2 (49) | 0.698 | 0.948 | 0.592 |
| *GCK1* | rs6952751 | 29.4±6.6 (500) | 30.2±9.1 (72) | 34.7±1.6 (3) | 0.281 | - | - |
| *GCK1* | rs735670 | 29.6±7.1 (524) | 28.9±5.7 (46) | 28.9±3.2 (5) | 0.530 | - | - |
| *IGFBP1* | rs4724445 | 29.6±6.9 (502) | 30.3±7.6 (63) | 27.5±2.2 (2) | 0.578 | - | - |
| *IGFBP1* | rs3763497 | 29.9±7.1 (394) | 29.2±7.1 (161) | 29.2±5.4 (12) | 0.293 | 0.309 | 0.814 |
| *IGFBP1* | rs1065780 | 30.4±6.5 (194) | 29.3±7.6 (279) | 29.5±5.9 (94) | 0.111 | 0.219 | 0.813 |
| *IGFBP1* | rs3828998 | 30.3±6.3 (188) | 29.3±7.7 (279) | 29.8±6.4 (100) | 0.167 | 0.376 | 0.927 |
| *IGFBP1* | rs3793344 | 30.3±6.5 (197) | 29.3±7.7 (274) | 29.5±5.9 (96) | 0.136 | 0.228 | 0.737 |
| *IGFBP1* | rs4619 | 30.3±6.7 (174) | 29.4±7.3 (282) | 29.5±6.7 (111) | 0.194 | 0.301 | 0.759 |
| *IGFBP1* | rs7454 | 29.8±7.1 (546) | 27.2±5.7 (18) | 22.3±0.0 (1) | 0.077 | - | - |
| *IGFBP1* | rs1908750 | 29.6±7.0 (531) | 31.4±7.3 (35) | 37.6±0.0 (1) | 0.108 | - | - |
| *IGFBP1* | rs9658231 | 29.7±7.1 (472) | 30.1±6.4 (90) | 21.3±2.8 (4) | 0.901 | - | **-** |
| *IGFBP1* | rs9658233 | 29.8±7.0 (383) | 29.6±7.0 (172) | 30.5±8.3 (11) | 0.855 | 0.930 | 0.751 |
| *IGFBP1* | rs9658238 | 29.9±7.1 (450) | 28.9±6.7 (113) | 27.5±3.9 (4) | 0.200 | - | - |
| *IGFBP1* | rs9658239 | 29.9±7.1 (527) | 27.6±5.6 (30) | 29.8±0.0 (1) | 0.106 | - | - |
| *IGFBP1* | rs1908751 | 29.8±7.3 (254) | 29.2±6.7 (243) | 31.6±7.1 (60) | 0.838 | 0.436 | **0.043** |
| *IGFBP1* | rs13442660 | 30.3±6.7 (176) | 29.5±7.3 (284) | 29.3±6.7 (107) | 0.209 | 0.233 | 0.527 |
| *IGFBP1* | rs1496495 | 29.9±6.8 (466) | 28.9±8.5 (79) | 29.9±6.2 (12) | 0.274 | 0.372 | 0.957 |
| *IGFBP1* | rs13441765 | 29.8±7.3 (307) | 29.6±6.5 (221) | 29.7±8.1 (38) | 0.732 | 0.796 | 0.967 |
| *IGFBP3* | rs12671484 | 29.8±7.1 (527) | 28.5±6.1 (37) | 29.8±0.0 (1) | 0.280 | - | - |
| *IGFBP3* | rs2270628 | 30.1±6.5 (217) | 29.5±7.5 (280) | 29.6±6.5 (70) | 0.345 | 0.459 | 0.934 |
| *IGFBP3* | rs13223993 | 30.1±6.5 (172) | 29.5±7.3 (276) | 29.7±7.2 (119) | 0.456 | 0.615 | 0.972 |
| *IGFBP3* | rs6670 | 29.7±7.2 (447) | 29.9±6.3 (114) | 26.9±7.8 (6) | 0.861 | - | - |
| *IGFBP3* | rs10255707 | 29.9±7.1 (511) | 27.3±5.4 (33) | 23.5±7.4 (2) | **0.022** | **-** | - |
| *IGFBP3* | rs3110697 | 30.1±6.8 (234) | 29.6±7.2 (270) | 28.3±7.2 (63) | 0.254 | 0.113 | 0.124 |
| *IGFBP3* | rs6953668 | 29.7±7.1 (494) | 29.6±6.7 (71) | 0 (0) | 0.900 | - | - |
| *IGFBP3* | rs2453837 | 29.8±7.1 (531) | 28.6±6.4 (33) | 27.5±0.0 (1) | 0.352 | - | - |
| *IGFBP3* | rs924140 | 30.2±7.3 (200) | 29.2±6.8 (276) | 29.3±7.0 (80) | 0.117 | 0.179 | 0.654 |
| *IGFBP3* | rs903889 | 29.9±7.1 (475) | 28.9±6.7 (91) | 25.9±0.0 (1) | 0.268 | - | - |
| *IGFBP3* | rs2453836 | 29.7±6.9 (356) | 29.5±7.1 (189) | 31.2±8.5 (21) | 0.907 | 0.835 | 0.358 |

1 denotes the major allele and 2 the minor allele. **Bold**: P-values <0.05.

**Supplementary Table 5** Nominally significant (P<0.05) Single-SNP genotypic tests of association with type 2 diabetic end-stage renal disease

| **Gene** | **Marker** | **Major†/Minor alleles** | **Dominant* P-value** | **Dominant Admixture P- value** | **OR**  **(95% CI)** | **Additive**  **P-value** | **Additive Admixture P-value** | **OR (95% CI)** | **Recessive P-value** | **Recessive Admixture P-value** | **OR (95% CI)** |
| --- | --- | --- | --- | --- | --- | --- | --- | --- | --- | --- | --- |
|  |  |  |  |  |  |  |  |  |  |  |  |
| *IGFBP1* | rs3763497 | G/A | 0.078 | 0.102 | 0.80 | **0.049** | 0.071 | 0.80 | 0.174 | 0.231 | 0.61 |
|  |  |  |  |  | (0.63-1.03) |  |  | (0.65-1.00) |  |  | (0.29-.25) |
| *IGFBP1* | rs9658233 | T/A | **0.021** | **0.03** | 1.35 | 0.055 | 0.069 | 1.25 | 0.592 | 0.658 | 0.80 |
|  |  |  |  |  | (1.05-1.75) |  |  | (0.99-1.57) |  |  | (0.36-.79) |
| *IGFBP3* | rs10255707 | G/A | **0.002** | **0.01** | 0.52 | - | - | - | - | - | - |
|  |  |  |  |  | (0.34-0.80) |  |  |  |  |  |  |
| *IGFBP3* | rs3110697 | G/A | 0.758 | 0.83 | 0.96 | 0.081 | 0.095 | 0.86 | **0.002** | **0.003** | 0.60 |
|  |  |  |  |  | (0.76-1.22) |  |  | (0.73-1.02) |  |  | (0.42-.84) |
| *IGFBP3* | rs924140 | A/G | 0.373 | 0.502 | 0.89 | **0.018** | **0.033** | 0.82 | **0.001** | **0.002** | 0.60 |
|  |  |  |  |  | (0.70-1.14) |  |  | (0.69-0.97) |  |  | (0.44-.82) |
| *IGFBP3* | rs903889 | A/C | **0.016** | **0.029** | 0.70 | - | - | - | - | - | - |
|  |  |  |  |  | (0.52-0.94) |  |  |  |  |  |  |

*Only the dominant model was considered where the minor allele homozygote count for either cases or controls was <10. Test models refer to the minor allele. †Major allele is defined as most common allele in controls. ‡Dominant model. **Bold**: Reported risk allele, and P-values <0.05.

**Supplementary Table 6. Single-SNP genotypic tests of association with type 2 diabetic end-stage renal disease**

| **Gene** | **Marker** | **Major†/Minor alleles** | **Dominant* P-value** | **Dominant Admixture P- value** | **OR (95% CI)** | **Additive P-value** | **Additive Admixture** | **OR (95% CI)** | **Recessive P-value** | **Recessive Admixture P-value** | **OR (95% CI)** |
| --- | --- | --- | --- | --- | --- | --- | --- | --- | --- | --- | --- |
|  |  |  |  |  |  |  | **P-value** |  |  |  |  |
| *IL-6* | rs12700386 | G/C | 0.785 | 0.762 | 1.04 (0.801.33) | 0.835 | 0.831 | 1.02 (0.821.28) | 0.917 | 0.862 | 0.96 (0.46-2.01) |
| *IL-6* | rs1800797 | G/A | 0.243 | 0.671 | 0.80 (0.561.16) | - | - | - | - | - | - |
| *IL-6* | rs1800796 | C/G | 0.312 | 0.406 | 1.16 (0.871.55) | - | - | - | - | - | - |
| *IL-6* | rs1800795 | C/G | 0.216 | 0.679 | 0.80 (0.561.14) | - | - | - | - | - | - |
| *IL-6* | rs2069830 | G/A | 0.694 | 0.612 | 0.94 (0.681.29) | - | - | - | - | - | - |
| *IL-6* | rs2069835 | A/G | 0.979 | 0.984 | 1.00 (0.751.35) | - | - | - | - | - | - |
| *IL-6* | rs1474347 | A/C | 0.655 | 0.355 | 1.06 (0.821.38) | 0.923 | 0.661 | 0.99 (0.791.23) | 0.159 | 0.306 | 0.62 (0.321.21) |
| *IL-6* | rs1524107 | G/A | 0.276 | 0.346 | 1.18 (0.871.60) | - | - | - | - | - | - |
| *IL-6* | rs2069840 | G/C | 0.673 | 0.928 | 0.95 (0.741.22) | 0.609 | 0.834 | 0.94 (0.761.18) | 0.646 | 0.672 | 0.85 (0.411.73) |
| *IL-6* | rs1554606 | C/A | 0.520 | 0.449 | 1.08 (0.861.36) | 0.501 | 0.420 | 1.06 (0.891.26) | 0.676 | 0.600 | 1.08 (0.751.55) |
| *IL-6* | rs2069842 | G/A | 0.513 | 0.660 | 1.11 (0.811.53) | - | - | - | - | - | - |
| *IL-6* | rs2069843 | G/A | 0.253 | 0.394 | 1.17 (0.891.55) | - | - | - | - | - | - |
| *IL-6* | rs2069860 | T/A | 0.968 | 0.576 | 1.03 (0.21-5.14) | - | - | - | - | - | - |
| *IL-6* | rs2069849 | G/A | 0.398 | 0.591 | 1.12 (0.861.45) | 0.318 | 0.497 | 1.12 (0.891.41) | 0.397 | 0.495 | 1.39 (0.65-2.96) |
| *IL-6* | rs2069861 | G/A | 0.663 | 0.874 | 0.86 (0.431.72) | - | - | - | - | - | - |
| *IL-6* | rs10242595 | A/G | 0.693 | 0.764 | 0.95 (0.741.23) | 0.622 | 0.455 | 1.04 (0.891.23) | 0.204 | 0.109 | 1.20 (0.911.58) |
| *IL-6* | rs11766273 | G/A | 0.221 | 0.353 | 0.66 (0.341.29) | - | - | - | - | - | - |
| *GCK1* | rs2908277 | G/A | 0.659 | 0.685 | 1.05 (0.841.33) | 0.745 | 0.816 | 1.03 (0.851.25) | 0.918 | 0.790 | 0.97 (0.591.60) |
| *GCK1* | rs2268576 | G/A | 0.747 | 0.941 | 0.96 (0.771.21) | 0.848 | 0.670 | 1.02 (0.851.22) | 0.300 | 0.262 | 1.25 (0.821.90) |
| *GCK1* | rs2268575 | A/G | 0.290 | 0.287 | 0.86 (0.661.13) | - | - | - | - | - | - |
| *GCK1* | rs2908296 | C/A | 0.193 | 0.188 | 1.17 (0.921.47) | 0.616 | 0.613 | 1.04 (0.881.24) | 0.342 | 0.334 | 0.84 (0.591.20) |
| *GCK1* | rs2971676 | G/A | 0.500 | 0.479 | 1.08 (0.861.37) | 0.826 | 0.813 | 1.02 (0.851.23) | 0.393 | 0.380 | 0.81 (0.491.32) |
| *GCK1* | rs2268572 | A/G | 0.856 | 0.980 | 0.98 (0.771.25) | 0.911 | 0.872 | 0.99 (0.841.16) | 0.989 | 0.796 | 1.00 (0.751.33) |
| *GCK1* | rs2971675 | C/A | 0.131 | 0.166 | 1.20 (0.951.52) | 0.247 | 0.313 | 1.12 (0.921.37) | 0.788 | 0.696 | 0.93 (0.551.58) |
| *GCK1* | rs758989 | A/G | 0.564 | 0.808 | 0.93 (0.731.19) | 0.855 | 0.866 | 0.98 (0.801.21) | 0.370 | 0.276 | 1.32 (0.72-2.44) |
| *GCK1* | rs2080033 | A/G | 0.914 | 0.699 | 1.01 (0.801.28) | 0.699 | 0.502 | 1.04 (0.861.26) | 0.431 | 0.353 | 1.23 (0.74-2.04) |
| *GCK1* | rs2971672 | C/A | 0.351 | 0.454 | 0.89 (0.701.14) | 0.538 | 0.669 | 0.95 (0.801.12) | 0.954 | 0.863 | 1.01 (0.741.39) |
| *GCK1* | rs12673242 | A/G | 0.844 | 0.892 | 1.02 (0.811.29) | 0.824 | 0.910 | 1.02 (0.861.20) | 0.871 | 0.974 | 1.03 (0.741.43) |
| *GCK1* | rs2908292 | G/A | 0.207 | 0.230 | 0.85 (0.671.09) | 0.297 | 0.330 | 0.89 (0.721.10) | 0.908 | 0.871 | 1.04 (0.551.95) |
| *GCK1* | rs2908290 | A/G | 0.783 | 0.836 | 0.97 (0.751.24) | 0.706 | 0.673 | 1.03 (0.881.21) | 0.336 | 0.337 | 1.15 (0.871.51) |
| *GCK1* | rs2284777 | A/G | 0.672 | 0.688 | 0.93 (0.661.31) | 0.753 | 0.762 | 0.96 (0.721.27) | 0.919 | 0.933 | 1.04 (0.45-2.43) |
| *GCK1* | rs2300586 | G/A | 0.610 | 0.639 | 1.06 (0.841.34) | 0.809 | 0.870 | 1.02 (0.851.23) | 0.689 | 0.611 | 0.91 (0.591.42) |
| *GCK1* | rs2300584 | A/G | 0.290 | 0.428 | 0.87 (0.671.13) | 0.333 | 0.495 | 0.89 (0.711.13) | 0.911 | 0.951 | 0.96 (0.43-2.11) |
| *GCK1* | rs758988 | C/A | 0.687 | 0.811 | 1.06 (0.811.39) | - | - | - | - | - | - |
| *GCK1* | rs758985 | G/A | 0.583 | 0.520 | 1.09 (0.791.51) | - | - | - | - | - | - |
| *GCK1* | rs1990458 | G/A | 0.300 | 0.497 | 0.88 (0.701.12) | 0.369 | 0.637 | 0.92 (0.751.11) | 0.908 | 0.810 | 0.97 (0.581.62) |
| *GCK1* | rs741038 | A/G | 0.676 | 0.668 | 1.05 (0.831.35) | 0.567 | 0.583 | 1.06 (0.871.28) | 0.541 | 0.593 | 1.16 (0.721.86) |
| *GCK1* | rs730497 | G/A | 0.567 | 0.492 | 1.07 (0.841.37) | 0.755 | 0.661 | 1.03 (0.851.26) | 0.659 | 0.722 | 0.88 (0.511.53) |
| *GCK1* | rs2908289 | G/A | 0.840 | 0.809 | 1.02 (0.811.29) | 0.579 | 0.561 | 1.05 (0.881.26) | 0.346 | 0.351 | 1.23 (0.801.89) |
| *GCK1* | rs6952751 | G/A | 0.880 | 0.995 | 1.03 (0.731.44) | - | - | - | - | - | - |
| *GCK1* | rs735670 | A/T | 0.605 | 0.584 | 0.90 (0.611.34) | - | - | - | - | - | - |
| *IGFBP1* | rs4724445 | G/A | 0.137 | 0.232 | 0.77 (0.551.09) | - | - | - | - | - | - |
| *IGFBP1* | rs3763497 | G/A | 0.078 | 0.102 | 0.80 (0.631.03) | **0.049** | 0.071 | 0.80 (0.651.00) | 0.174 | 0.231 | 0.61 (0.291.25) |
| *IGFBP1* | rs1065780 | G/A | 0.399 | 0.411 | 1.11 (0.871.41) | 0.760 | 0.692 | 1.03 (0.871.21) | 0.615 | 0.757 | 0.92 (0.681.26) |
| *IGFBP1* | rs3828998 | A/G | 0.498 | 0.522 | 1.09 (0.851.39) | 0.912 | 0.860 | 1.01 (0.861.19) | 0.530 | 0.643 | 0.91 (0.671.23) |
| *IGFBP1* | rs3793344 | A/G | 0.437 | 0.442 | 1.10 (0.861.40) | 0.731 | 0.655 | 1.03 (0.871.21) | 0.731 | 0.886 | 0.95 (0.701.29) |
| *IGFBP1* | rs4619 | A/G | 0.307 | 0.340 | 1.14 (0.891.46) | 0.643 | 0.652 | 1.04 (0.881.22) | 0.713 | 0.755 | 0.95 (0.711.26) |
| *IGFBP1* | rs7454 | G/C | 0.260 | 0.149 | 1.49 (0.74-2.97) | - | - | - | - | - | - |
| *IGFBP1* | rs1908750 | G/A | 0.261 | 0.360 | 1.33 (0.81-2.20) | - | - | - | - | - | - |
| *IGFBP1* | rs9658231 | C/A | 0.162 | 0.191 | 0.81 (0.601.09) | - | - | - | - | - | - |
| *IGFBP1* | rs9658233 | T/A | **0.021** | **0.030** | 1.35 (1.051.75) | 0.055 | 0.069 | 1.25 (0.991.57) | 0.592 | 0.658 | 0.80 (0.361.79) |
| *IGFBP1* | rs9658238 | A/G | 0.937 | 0.862 | 1.01 (0.761.35) | - | - | - | - | - | - |
| *IGFBP1* | rs9658239 | A/G | 0.305 | 0.505 | 0.78 (0.481.26) | - | - | - | - | - | - |
| *IGFBP1* | rs1908751 | C/T | 0.148 | 0.184 | 1.19 (0.941.50) | 0.345 | 0.361 | 1.09 (0.911.29) | 0.783 | 0.877 | 0.95 (0.651.38) |
| *IGFBP1* | rs13442660 | A/C | 0.309 | 0.339 | 1.14 (0.891.46) | 0.530 | 0.531 | 1.05 (0.891.24) | 0.931 | 0.986 | 0.99 (0.731.33) |
| *IGFBP1* | rs1496495 | T/C | 0.511 | 0.635 | 0.90 (0.661.23) | - | - | - | - | - | - |
| *IGFBP1* | rs13441765 | A/C | 0.626 | 0.662 | 1.06 (0.841.34) | 0.433 | 0.476 | 1.08 (0.891.31) | 0.323 | 0.362 | 1.28 (0.78-2.09) |
| *IGFBP3* | rs12671484 | A/G | 0.067 | 0.106 | 0.67 (0.441.03) | - | - | - | - | - | - |
| *IGFBP3* | rs2270628 | G/A | 0.083 | 0.116 | 1.23 (0.971.56) | 0.321 | 0.368 | 1.09 (0.921.29) | 0.593 | 0.624 | 0.91 (0.641.29) |
| *IGFBP3* | rs13223993 | G/A | 0.088 | 0.126 | 1.24 (0.971.59) | 0.174 | 0.236 | 1.12 (0.951.31) | 0.657 | 0.742 | 1.07 (0.801.42) |
| *IGFBP3* | rs6670 | A/T | 0.056 | 0.097 | 0.77 (0.581.01) | - | - | - | - | - | - |
| *IGFBP3* | rs10255707 | G/A | **0.002** | **0.010** | 0.52 (0.34-0.80) | - | - | - | - | - | - |
| *IGFBP3* | rs3110697 | G/A | 0.758 | 0.830 | 0.96 (0.761.22) | 0.081 | 0.095 | 0.86 (0.731.02) | **0.002** | **0.003** | 0.60 (0.42-0.84) |
| *IGFBP3* | rs6953668 | G/A | 0.707 | 0.473 | 0.94 (0.661.32) | - | - | - | - | - | - |
| *IGFBP3* | rs2453837 | G/A | 0.903 | 0.903 | 0.97 (0.601.56) | - | - | - | - | - | - |
| *IGFBP3* | rs924140 | A/G | 0.373 | 0.502 | 0.89 (0.701.14) | **0.018** | **0.033** | 0.82 (0.69-0.97) | **0.001** | **0.002** | 0.60 (0.44-0.82) |
| *IGFBP3* | rs903889 | A/C | **0.016** | **0.029** | 0.70 (0.52-0.94) | - | - | - | - | - | - |
| *IGFBP3* | rs2453836 | A/G | 0.398 | 0.409 | 1.11 (0.871.41) | 0.701 | 0.694 | 1.04 (0.851.27) | 0.356 | 0.399 | 0.76 (0.431.36) |

Test models refer to the minor allele. *Only the dominant model was considered where the minor allele homozygote count was <10. **Bold**: P-values <0.05.

**Supplementary Table7**. Genotype frequencies in African American case and control groups

| **Gene** | **Marker** | **Location*** | **Major†/ Minor** | **T2D-ESRD Cases**  **Frequency (n)** | | | **Controls**  **Frequency (n)** | | |
| --- | --- | --- | --- | --- | --- | --- | --- | --- | --- |
|  |  |  | **alleles** | **1/1** | **1/2** | **2/2** | **1/1** | **1/2** | **2/2** |
| *IL6* | rs12700386 | 22536249 | G/C | 0.706 (406) | 0.270 (155) | 0.024 (14) | 0.713 (423) | 0.261 (155) | 0.025 (15) |
| *IL6* | rs1800797 | 22539461 | G/A | 0.898 (508) | 0.099 (56) | 0.004 (2) | 0.876 (507) | 0.116 (67) | 0.009 (5) |
| *IL6* | rs1800796 | 22539486 | C/G | 0.791 (454) | 0.206 (118) | 0.003 (2) | 0.815 (483) | 0.177 (105) | 0.008 (5) |
| *IL6* | rs1800795 | 22539885 | C/G | 0.890 (512) | 0.106 (61) | 0.003 (2) | 0.867 (514) | 0.123 (73) | 0.010 (6) |
| *IL6* | rs2069830 | 22540377 | G/A | 0.848 (485) | 0.142 (81) | 0.010 (6) | 0.840 (497) | 0.152 (90) | 0.008 (5) |
| *IL6* | rs2069835 | 22541111 | A/G | 0.814 (468) | 0.172 (99) | 0.014 (8) | 0.815 (483) | 0.174 (103) | 0.012 (7) |
| *IL6* | rs1474347 | 22541364 | A/C | 0.732 (421) | 0.243 (140) | 0.024 (14) | 0.744 (441) | 0.218 (129) | 0.039 (23) |
| *IL6* | rs1524107 | 22541459 | G/A | 0.816 (469) | 0.181 (104) | 0.003 (2) | 0.840 (498) | 0.157 (93) | 0.003 (2) |
| *IL6* | rs2069840 | 22541812 | G/C | 0.706 (406) | 0.270 (155) | 0.024 (14) | 0.695 (412) | 0.277 (164) | 0.029 (17) |
| *IL6* | rs1554606 | 22541947 | C/A | 0.449 (255) | 0.431 (245) | 0.120 (68) | 0.468 (276) | 0.420 (248) | 0.112 (66) |
| *IL6* | rs2069842 | 22542550 | G/A | 0.839 (481) | 0.155 (89) | 0.005 (3) | 0.853 (506) | 0.142 (84) | 0.005 (3) |
| *IL6* | rs2069843 | 22543234 | G/A | 0.750 (414) | 0.234 (129) | 0.016 (9) | 0.779 (444) | 0.212 (121) | 0.009 (5) |
| *IL6* | rs2069860 | 22544278 | T/A | 0.997 (572) | 0.003 (2) | 0 (0) | 0.997 (591) | 0.003 (2) | 0 (0) |
| *IL6* | rs2069849 | 22544396 | G/A | 0.729 (419) | 0.243 (140) | 0.028 (16) | 0.750 (445) | 0.229 (136) | 0.020 (12) |
| *IL6* | rs2069861 | 22544894 | G/A | 0.976 (560) | 0.024 (14) | 0 (0) | 0.971 (576) | 0.029 (17) | 0 (0) |
| *IL6* | rs10242595 | 22547471 | A/G | 0.287 (165) | 0.480 (276) | 0.233 (134) | 0.277 (164) | 0.521 (309) | 0.202 (120) |
| *IL6* | rs11766273 | 22548903 | G/A | 0.976 (561) | 0.024 (14) | 0 (0) | 0.963 (571) | 0.037 (22) | 0 (0) |
| *GCK1* | rs2908277 | 43956673 | G/A | 0.541 (311) | 0.403 (232) | 0.056 (32) | 0.554 (330) | 0.389 (232) | 0.057 (34) |
| *GCK1* | rs2268576 | 43962263 | G/A | 0.506 (291) | 0.403 (232) | 0.090 (52) | 0.497 (296) | 0.430 (256) | 0.074 (44) |
| *GCK1* | rs2268575 | 43962514 | A/G | 0.773 (443) | 0.216 (124) | 0.010 (6) | 0.747 (445) | 0.232 (138) | 0.022 (13) |
| *GCK1* | rs2908296 | 43966702 | C/A | 0.409 (235) | 0.482 (277) | 0.110 (63) | 0.446 (266) | 0.426 (254) | 0.128 (76) |
| *GCK1* | rs2971676 | 43967722 | G/A | 0.570 (328) | 0.377 (217) | 0.052 (30) | 0.590 (351) | 0.346 (206) | 0.064 (38) |
| *GCK1* | rs2268572 | 43967791 | A/G | 0.332 (191) | 0.468 (269) | 0.200 (115) | 0.327 (195) | 0.473 (282) | 0.200 (119) |
| *GCK1* | rs2971675 | 43968078 | C/A | 0.588 (338) | 0.365 (210) | 0.047 (27) | 0.631 (376) | 0.319 (190) | 0.050 (30) |
| *GCK1* | rs758989 | 43976246 | A/G | 0.685 (394) | 0.273 (157) | 0.042 (24) | 0.669 (399) | 0.299 (178) | 0.032 (19) |
| *GCK1* | rs2080033 | 43977562 | A/G | 0.609 (350) | 0.332 (191) | 0.059 (34) | 0.612 (364) | 0.339 (202) | 0.049 (29) |
| *GCK1* | rs2971672 | 43979146 | C/A | 0.371 (208) | 0.469 (263) | 0.160 (90) | 0.344 (199) | 0.497 (287) | 0.159 (92) |
| *GCK1* | rs12673242 | 43980734 | A/G | 0.410 (236) | 0.449 (258) | 0.141 (81) | 0.416 (248) | 0.446 (266) | 0.138 (82) |
| *GCK1* | rs2908292 | 43983950 | G/A | 0.694 (399) | 0.271 (156) | 0.035 (20) | 0.659 (393) | 0.307 (183) | 0.034 (20) |
| *GCK1* | rs2908290 | 43989377 | A/G | 0.304 (175) | 0.468 (269) | 0.228 (131) | 0.297 (177) | 0.498 (297) | 0.205 (122) |
| *GCK1* | rs2284777 | 43989838 | A/G | 0.877 (497) | 0.104 (59) | 0.019 (11) | 0.868 (514) | 0.113 (67) | 0.019 (11) |
| *GCK1* | rs2300586 | 43992096 | G/A | 0.506 (291) | 0.424 (244) | 0.070 (40) | 0.521 (310) | 0.403 (240) | 0.076 (45) |
| *GCK1* | rs2300584 | 43992578 | A/G | 0.762 (438) | 0.217 (125) | 0.021 (12) | 0.735 (438) | 0.243 (145) | 0.022 (13) |
| *GCK1* | rs758988 | 43994821 | C/A | 0.767 (441) | 0.214 (123) | 0.019 (11) | 0.777 (463) | 0.208 (124) | 0.015 (9) |
| *GCK1* | rs758985 | 43995711 | G/A | 0.845 (486) | 0.139 (80) | 0.016 (9) | 0.857 (508) | 0.137 (81) | 0.007 (4) |
| *GCK1* | rs1990458 | 43996042 | G/A | 0.607 (349) | 0.341 (196) | 0.052 (30) | 0.577 (344) | 0.369 (220) | 0.054 (32) |
| *GCK1* | rs741038 | 43996719 | A/G | 0.658 (374) | 0.273 (155) | 0.069 (39) | 0.670 (392) | 0.270 (158) | 0.060 (35) |
| *GCK1* | rs730497 | 43996961 | G/A | 0.642 (367) | 0.315 (180) | 0.044 (25) | 0.658 388) | 0.293 (173) | 0.049 (29) |
| *GCK1* | rs2908289 | 43997182 | G/A | 0.548 (315) | 0.367 (211) | 0.085 (49) | 0.554 (330) | 0.376 (224) | 0.070 (42) |
| *GCK1* | rs6952751 | 43999953 | G/A | 0.870 (500) | 0.125 (72) | 0.005 (3) | 0.872 (520) | 0.126 (75) | 0.002 (1) |
| *GCK1* | rs735670 | 44006585 | A/T | 0.911 (524) | 0.080 (46) | 0.009 (5) | 0.903 (537) | 0.094 (56) | 0.003 (2) |
| *IGFBP1* | rs4724445 | 45696440 | G/A | 0.885 (502) | 0.111 (63) | 0.004 (2) | 0.856 (498) | 0.143 (83) | 0.002 (1) |
| *IGFBP1* | rs3763497 | 45698588 | G/A | 0.695 (394) | 0.284 (161) | 0.021 (12) | 0.646 (376) | 0.320 (186) | 0.034 (20) |
| *IGFBP1* | rs1065780 | 45700917 | G/A | 0.342 (194) | 0.492 (279) | 0.166 (94) | 0.366 (213) | 0.457 (266) | 0.177 (103) |
| *IGFBP1* | rs3828998 | 45702048 | A/G | 0.332 (188) | 0.492 (279) | 0.176 (100) | 0.351 (204) | 0.459 (267) | 0.191 (111) |
| *IGFBP1* | rs3793344 | 45702135 | A/G | 0.347 (197) | 0.483 (274) | 0.169 (96) | 0.369 (215) | 0.454 (264) | 0.177 (103) |
| *IGFBP1* | rs4619 | 45705909 | A/G | 0.307 (174) | 0.497 (282) | 0.196 (111) | 0.335 (195) | 0.460 (268) | 0.204 (119) |
| *IGFBP1* | rs7454 | 45706246 | G/C | 0.966 (546) | 0.032 (18) | 0.002 (1) | 0.978 (568) | 0.022 (13) | 0 (0) |
| *IGFBP1* | rs1908750 | 45706320 | G/A | 0.937 (531) | 0.062 (35) | 0.002 (1) | 0.952 (554) | 0.046 (27) | 0.002 (1) |
| *IGFBP1* | rs9658231 | 45706664 | C/A | 0.834 (472) | 0.159 (90) | 0.007 (4) | 0.802 (466) | 0.189 (110) | 0.009 (5) |
| *IGFBP1* | rs9658233 | 45706981 | T/A | 0.677 (383) | 0.304 (172) | 0.019 (11) | 0.739 (430) | 0.237 (138) | 0.024 (14) |
| *IGFBP1* | rs9658238 | 45707379 | A/G | 0.794 (450) | 0.199 (113) | 0.007 (4) | 0.796 (463) | 0.198 (115) | 0.007 (4) |
| *IGFBP1* | rs9658239 | 45707399 | A/G | 0.944 (527) | 0.054 (30) | 0.002 (1) | 0.929 (526) | 0.067 (38) | 0.004 (2) |
| *IGFBP1* | rs1908751 | 45708759 | C/T | 0.456 (254) | 0.436 (243) | 0.108 (60) | 0.499 (283) | 0.388 (220) | 0.113 (64) |
| *IGFBP1* | rs13442660 | 45708931 | A/C | 0.310 (176) | 0.501 (284) | 0.189 (107) | 0.338 (197) | 0.471 (274) | 0.191 (111) |
| *IGFBP1* | rs1496495 | 45710501 | T/C | 0.837 (466) | 0.142 (79) | 0.022 (12) | 0.822 (466) | 0.173 (98) | 0.005 (3) |
| *IGFBP1* | rs13441765 | 45711264 | A/C | 0.542 (307) | 0.390 (221) | 0.067 (38) | 0.557 (324) | 0.390 (227) | 0.053 (31) |
| *IGFBP3* | rs12671484 | 45720395 | A/G | 0.933 (527) | 0.065 (37) | 0.002 (1) | 0.903 (519) | 0.094 (54) | 0.003 (2) |
| *IGFBP3* | rs2270628 | 45722810 | G/A | 0.383 (217) | 0.494 (280) | 0.123 (70) | 0.433 (252) | 0.433 ( 252) | 0.134 (78) |
| *IGFBP3* | rs13223993 | 45724470 | G/A | 0.303 (172) | 0.487 (276) | 0.210 (119) | 0.351 (204) | 0.450 (262) | 0.199 (116) |
| *IGFBP3* | rs6670 | 45725494 | A/T | 0.788 (447) | 0.201 (114) | 0.011 (6) | 0.741 (431) | 0.232 (135) | 0.027 (16) |
| *IGFBP3* | rs10255707 | 45727932 | G/A | 0.936 (511) | 0.060 (33) | 0.004 (2) | 0.883 (484) | 0.108 (59) | 0.009 (5) |
| *IGFBP3* | rs3110697 | 45728269 | G/A | 0.413 (234) | 0.476 (270) | 0.111 (63) | 0.404 (235) | 0.423 (246) | 0.174 (101) |
| *IGFBP3* | rs6953668 | 45729115 | G/A | 0.874 (494) | 0.126 (71) | 0 (0) | 0.867 (501) | 0.131 (76) | 0.002 (1) |
| *IGFBP3* | rs2453837 | 45729290 | G/A | 0.940 (531) | 0.058 (33) | 0.002 (1) | 0.938 (545) | 0.062 (36) | 0 (0) |
| *IGFBP3* | rs924140 | 45736354 | A/G | 0.360 (200) | 0.496 (276) | 0.144 (80) | 0.334 (193) | 0.447 (258) | 0.218 (126) |
| *IGFBP3* | rs903889 | 45738235 | A/C | 0.838 (475) | 0.160 (91) | 0.002 (1) | 0.782 (455) | 0.210 (122) | 0.009 (5) |
| *IGFBP3* | rs2453836 | 45739035 | A/G | 0.629 (356) | 0.334 (189) | 0.037 (21) | 0.653 (380) | 0.299 (174) | 0.048 (28) |

*NCBI Build 36.3 (August 2009). †Major allele is defined as most common allele in controls.
